# Genetic Analysis of the Neurosteroid Deoxycorticosterone and Its Relation to Alcohol Phenotypes: Identification of QTLs and Downstream Gene Regulation

**DOI:** 10.1371/journal.pone.0018405

**Published:** 2011-04-08

**Authors:** Patrizia Porcu, Todd K. O'Buckley, Soomin C. Song, Jo Lynne Harenza, Lu Lu, Xusheng Wang, Robert W. Williams, Michael F. Miles, A. Leslie Morrow

**Affiliations:** 1 Department of Psychiatry, University of North Carolina School of Medicine, Chapel Hill, North Carolina, United States of America; 2 Bowles Center for Alcohol Studies, University of North Carolina School of Medicine, Chapel Hill, North Carolina, United States of America; 3 Departments of Neurology and Pharmacology/Toxicology and The Center for the Study of Biological Complexity, Virginia Commonwealth University, Richmond, Virginia, United States of America; 4 Department of Anatomy and Neurobiology and Center for Integrative and Translational Genomics, University of Tennessee Health Science Center, Memphis, Tennessee, United States of America; 5 Department of Pharmacology, University of North Carolina School of Medicine, Chapel Hill, North Carolina, United States of America; The University of Hong Kong, Hong Kong

## Abstract

**Background:**

Deoxycorticosterone (DOC) is an endogenous neurosteroid found in brain and serum, precursor of the GABAergic neuroactive steroid (3α,5α)-3,21-dihydroxypregnan-20-one (tetrahydrodeoxycorticosterone, THDOC) and the glucocorticoid corticosterone. These steroids are elevated following stress or ethanol administration, contribute to ethanol sensitivity, and their elevation is blunted in ethanol dependence.

**Methodology/Principal Findings:**

To systematically define the genetic basis, regulation, and behavioral significance of DOC levels in plasma and cerebral cortex we examined such levels across 47 young adult males from C57BL/6J (B6)×DBA/2J (D2) (BXD) mouse strains for quantitative trait loci (QTL) and bioinformatics analyses of behavior and gene regulation. Mice were injected with saline or 0.075 mg/kg dexamethasone sodium salt at 8:00 am and were sacrificed 6 hours later. DOC levels were measured by radioimmunoassay. Basal cerebral cortical DOC levels ranged between 1.4 and 12.2 ng/g (8.7-fold variation, *p*<0.0001) with a heritability of ∼0.37. Basal plasma DOC levels ranged between 2.8 and 12.1 ng/ml (4.3-fold variation, *p*<0.0001) with heritability of ∼0.32. QTLs for basal DOC levels were identified on chromosomes 4 (cerebral cortex) and 14 (plasma). Dexamethasone-induced changes in DOC levels showed a 4.4-fold variation in cerebral cortex and a 4.1-fold variation in plasma, but no QTLs were identified. DOC levels across BXD strains were further shown to be co-regulated with networks of genes linked to neuronal, immune, and endocrine function. DOC levels and its responses to dexamethasone were associated with several behavioral measures of ethanol sensitivity previously determined across the BXD strains by multiple laboratories.

**Conclusions/Significance:**

Both basal and dexamethasone-suppressed DOC levels are positively correlated with ethanol sensitivity suggesting that the neurosteroid DOC may be a putative biomarker of alcohol phenotypes. DOC levels were also strongly correlated with networks of genes associated with neuronal function, innate immune pathways, and steroid metabolism, likely linked to behavioral phenotypes.

## Introduction

Deoxycorticosterone (DOC) is an endogenous neurosteroid present in the brain as well as in the peripheral circulation. It is synthesized from progesterone, mainly in the adrenal zona fasciculata and it is precursor of both the glucocorticoid corticosterone and the GABAergic neuroactive steroid (3α,5α)-3,21-dihydroxypregnan-20-one (tetrahydrodeoxycorticosterone, THDOC). These steroids are all elevated following acute stress [1] or ethanol administration in rats, and their elevation is blunted in ethanol dependence [2], [3], [4]. The ethanol-induced increases in neurosteroid levels are mediated by the hypothalamic-pituitary-adrenal (HPA) axis, since they are no longer observed following adrenalectomy/gonadectomy [5], [6], [7] or hypophysectomy [8]. Indeed, HPA axis regulation of neurosteroids appears to be critical in several neuropsychiatric disorders including alcoholism. DOC levels are regulated by hypothalamic and pituitary activation of the HPA axis in both cynomolgus monkeys and humans, and this regulation is altered following ethanol dependence [9], [10]. Furthermore, we have found that dexamethasone suppression of plasma DOC levels predicted subsequent voluntary alcohol consumption in ethanol-naïve cynomolgus monkeys [9]. That is, ethanol-naïve monkeys that are insensitive to dexamethasone drink the most alcohol in a two-bottle self-administration paradigm, suggesting that DOC responses may be putative biomarkers of excessive drinking phenotypes.

Individual differences in vulnerability to alcoholism have a genetic component [11], [12], [13], [14]. Furthermore, studies in rodents indicate a shared genetic sensitivity to ethanol, anxiety, and stress/HPA axis response [15], [16], [17]. Inbred mice are an excellent population model to study genetic and phenotypic variability. In particular, the C57BL/6J (B6)×DBA/2J (D2) (BXD) recombinant inbred strains have proved to be an extremely valuable reference population to study networks of phenotypes and their modulation by gene variants [18], [19], [20], [21]. The parental strains, B6 and D2, have been sequenced, and approximately five million single nucleotide polymorphisms (SNPs) between them have been identified. Several behavioral phenotypes for ethanol and stress/anxiety have already been characterized across the BXD strains by several independent labs and data is publicly available in GeneNetwork (www.genenetwork.org), a public repository of genetic and phenotypic data as well as a resource for multivariate genetic analysis of complex traits in genetic reference populations [22], [23], [24], [25].

In the present study we systematically defined genetic variation in basal levels of the neurosteroid DOC and dexamethasone-induced DOC responses across the BXD strains. We further analyzed genetic correlations between DOC levels and genetic or phenotypic data previously determined in the BXD panel by multiple independent laboratories and available in GeneNetwork.

## Results

### Basal DOC levels in BXD strains

**Figure 1:**
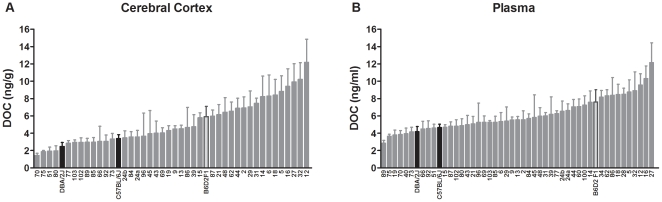
Variation in basal DOC levels across the BXD strains. Mice were injected with saline at 8:00 am and were sacrificed 6 hours later. Data are expressed as ng/g (cerebral cortex) or ng/ml (plasma) and are means ± SEM of values from 2–9 mice per strain. The *x* axis reports the BXD strain number; C57BL/6J (B6), DBA/2J (D2) and B6D2 F1 hybrid are also indicated. Strains are plotted in order from the lowest to the highest DOC levels for cerebral cortex (42 BXD strains) or plasma (47 BXD strains), respectively. One-way ANOVA was used to estimate significant genetic variation.

**Figure 2:**
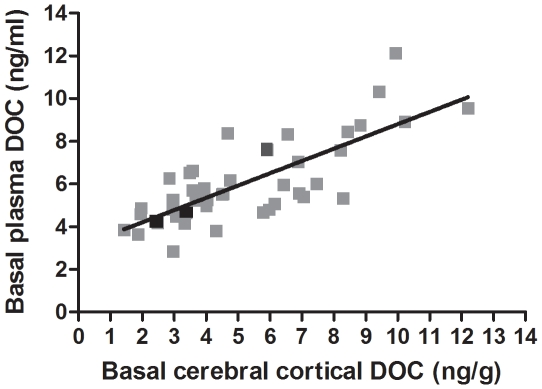
Correlation between cerebral cortical vs. plasma basal DOC levels across the BXD strains. DOC levels are expressed in ng/g (cerebral cortex) or ng/ml (plasma). Gray squares represent BXD strains, black squares are C57BL/6J (B6) and DBA/2J (D2) strains and dark gray square is the B6D2 F1 hybrid. Pearson *r* = 0.78, *p*<0.0001; Spearman *r* = 0.74, *p*<0.0001; *n* = 43.

We examined basal DOC levels across the BXD strains, including their parental strains and the B6D2 F1 hybrid. The data was obtained from the saline-treated mice. There was significant genetic variation in basal DOC levels in both cerebral cortex and plasma, as revealed by comparison of basal DOC levels across all the strains by one-way ANOVA. Figure 1a shows cerebral cortical DOC levels across the BXD strains examined (*n* = 42) as well as, the B6D2 F1 and the parental strains. Values range between 1.4 and 12.2 ng/g resulting in 8.7-fold genetic variation [F_(43,246)_ = 4.33, *p*<0.0001] of this trait. Heritability (*h^2^*) for this trait was estimated to be approximately 0.37 using a conservative measure (the intraclass correlation, see http://www.genenetwork.org/glossary.html#H) or ∼0.68 using the Hegmann and Possidente's method [26]. Basal plasma DOC levels across the BXD strains examined (*n* = 47), the B6D2 F1 hybrid and the parental strains range between 2.8 and 12.1 ng/ml, resulting in a 4.3-fold genetic variation [F_(48,282)_ = 3.69, *p*<0.0001] (Figure 1b). Heritability (*h^2^*) for this trait was estimated to be ∼0.32 using the intraclass correlation and ∼0.65 using the Hegmann and Possidente's method. The similar pattern of variation resulted in a positive correlation between plasma vs. cerebral cortical basal DOC levels across these strains (Pearson *r* = 0.78, *p*<0.0001; Spearman *r* = 0.74, p<0.0001, *n* = 43 strains, including parents and F1 hybrids; figure 2). This indicates that approximately 60% of the variance is shared between these traits and that as much as 40% is unique to each tissue source.

### Genetic correlations with behavioral phenotypes across the BXD strains

**Figure 3:**
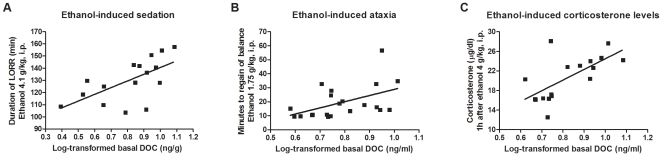
Genetic correlations between basal cerebral cortical or plasma DOC levels and behavioral phenotypes for ethanol sensitivity previously characterized in the BXD strains. Behavioral data for ethanol sensitivity has been collected by multiple independent labs and has been obtained from GeneNetwork (www.genenetwork.org). A) Pearson's *r* = 0.63, *p* = 0.008, Spearman's *r* = 0.64, *p* = 0.006, *n* = 16; Rodriguez *et al.*, 1994, GN ID 10586. B) Pearson's *r* = 0.49, *p* = 0.024, Spearman's *r* = 0.57, *p* = 0.006, *n* = 21; Kirstein *et al.*, 2002, GN ID 10350. C) Pearson's *r* = 0.67, *p* = 0.003, Spearman's *r* = 0.73, *p* = 0.0005, *n* = 17; Roberts *et al.*, 1995, GN ID 10573.

**Figure 4:**
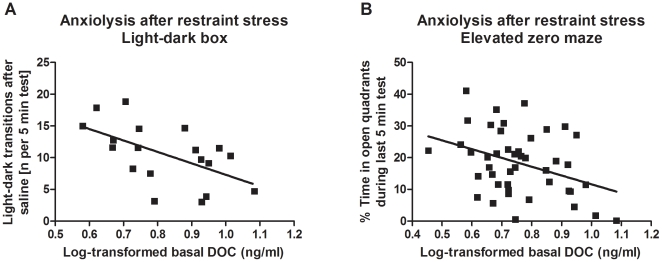
Genetic correlations between basal plasma DOC levels and phenotypes for anxiety-like behavior previously characterized in the BXD strains. Data for anxiety-like behavior has been collected by two independent labs and has been obtained from GeneNetwork (www.genenetwork.org). A) Pearson's *r* = −0.56, *p* = 0.011, Spearman's *r* = −0.63, *p* = 0.003, *n* = 19; Putman & Miles, manuscript submitted, GN ID 10960. B) Pearson's *r* = −0.37, *p* = 0.012, Spearman's *r* = −0.34, *p* = 0.022, *n* = 44; Cook *et al.*, unpublished, GN ID 12467.

Variation in basal DOC levels in both plasma and cerebral cortex was linked to several ethanol and anxiety phenotypes previously characterized across the BXD strains by other laboratories whose data are available in GeneNetwork (Figures 3 and 4). Basal DOC levels are positively correlated with increased ethanol-induced sedation (Pearson's *r* = 0.63, *p* = 0.008, Spearman's *r* = 0.64, *p* = 0.006, *n* = 16; [27]), ethanol-induced ataxia (Pearson's *r* = 0.49, *p* = 0.024, Spearman's *r* = 0.57, *p* = 0.006, *n* = 21; [28]), and ethanol-induced corticosterone levels (Pearson's *r* = 0.67, *p* = 0.003, Spearman's *r* = 0.73, *p* = 0.0005, *n* = 17; [29]) (Figure 3).

Correlations between basal plasma DOC levels and anxiety-associated phenotypes are moderately high (Figure 4). Basal DOC levels are associated with anxiety-like behaviors, measured 15 minutes after restraint stress in the light/dark box test (Pearson's *r* = −0.56, *p* = 0.011, Spearman's *r* = −0.63, *p* = 0.003, *n* = 19; Putman & Miles, manuscript submitted, GN ID: 10960), and in the elevated zero maze (Pearson's *r* = −0.37, *p* = 0.012, Spearman's *r* = −0.34, *p* = 0.022, *n* = 44; Cook et al., unpublished, GN ID: 12467).

Other relevant correlations and a cluster map for behavioral traits are reported in Table S1 and Figure S1, respectively. Interestingly, both cerebral cortical and plasma basal DOC levels were correlated with adrenal weight (GN ID: 11299) [30], which is important because adrenals are the major source of DOC production. Basal DOC levels are also correlated with behavioral measures of morphine and cocaine sensitivity, seizures susceptibility, pain sensitivity, sweet/bitter taste responses, number of tyrosine hydroxylase neurons, D1 dopamine receptor receptors (DRD1) in several brain regions, dopamine transporter SLC6A3 density in prefrontal cortex, and the number of 5-bromo-2′-deoxyuridine (BrdU)-labeled cells, a measure of adult neurogenesis.

### Mapping the QTLs for DOC

**Figure 5:**
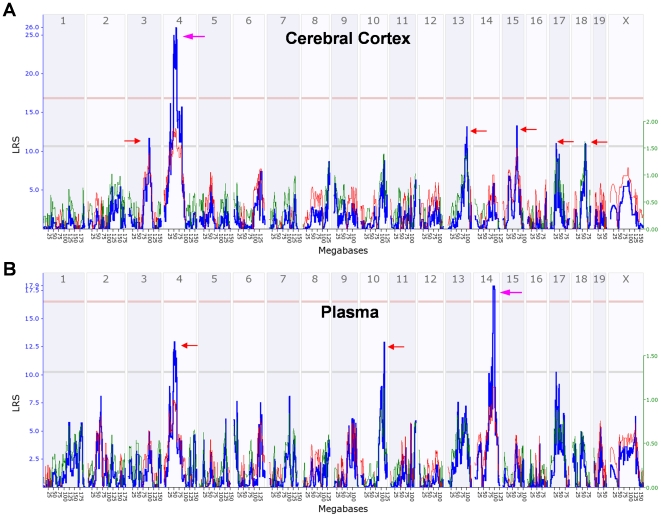
Genome-wide interval mapping plot for basal cerebral cortical and plasma DOC levels across the BXD strains and their parental strains. Mice were injected with saline at 8:00 am and were sacrificed 6 hours later. A) Likelihood ratio statistic (LRS) scores for basal cerebral cortical DOC levels across the entire genome show a significant QTL on chromosome 4 (purple arrow) and suggestive QTLs on chromosomes 3, 13, 15, 17 and 18 (red arrows). B) LRS scores for basal plasma DOC levels across the entire genome show a significant QTL on chromosome 14 (purple arrow) and suggestive QTLs on chromosomes 4, 10 and 17 (red arrows). The y axis and the thick blue lines provide the LRS of the association between the trait and the genotypes of markers. The two horizontal lines are the suggestive (gray) and significance (red) thresholds computed using 1000 permutations. A positive additive coefficient (green line) indicates that *D* alleles increase trait values. A negative additive coefficient (red line) indicates that *B* alleles increase trait values.

Variation in basal cerebral cortical DOC levels across the BXD strains was mapped using genetic and bioinformatics tools in GeneNetwork. A significant quantitative trait locus (QTL) was detected on chromosome 4 with a peak at 60 Mb (support interval ∼46–63 Mb), a likelihood ratio statistic (LRS) of 29 and a high *B* allele (Figure 5a). Suggestive loci mapped on chromosomes 3 (*B* allele high), 13 (*D* allele high), 15 (*B* allele high), 17 (*D* allele high) and 18 (*D* allele high). Basal DOC levels in cerebral cortex and in plasma have substantial shared correlation, but the overlap of QTLs was modest. Corresponding mapping for basal plasma DOC levels revealed a significant QTL on chromosome 14 between 93 and 100 Mb, (LRS of 19 and a high *B* allele) and three suggestive QTLs on chromosomes 4 (*B* allele high), 10 (*D* allele high), and 17 (*D* allele high) (Figure 5b). One of these suggestive loci, that on chromosome 4, precisely overlapped the significant locus for basal cortical DOC levels. The suggestive loci on chromosome 17 for both DOC phenotypes have the same location near the major histocompatibility complex, the same effect size (∼1 ng/g per allele), and the same polarity (high *D* alleles).

We used extensive available information regarding sequence variants between the B6 and D2 progenitor strains and gene expression data across the BXD panel to identify potential candidate genes for the two significant QTLs on chromosomes 4 and 14. The chromosome 4 interval contained ∼108 genes (GeneNetwork Interval Analyst, UCSC mm9 database) of which, 16 contained SNPs that distinguish between *B* and *D* haplotypes and that are predicted to produce non-conservative amino acid changes (http://genenetwork.org/webqtl/main.py?FormID=snpBrowser) (Table S2). Analysis of these variants using PolyPhen highlighted eight SNPs in six genes (*Nipsnap3b*, *Zfp462*, *D730040F13Rik*, *Zkscan16*, *Susd1* and *Slc46a2*) that are likely to be deleterious (Table S2). Additionally, analysis of BXD expression data from whole brain, prefrontal cortex, and liver identified significant *cis*-acting expression QTLs (*cis*-eQTLs) in 11 genes within the chromosome 4 support interval (Table S3). The chromosome 14 QTL contained only 26 genes of which 5 had *B* vs. *D* non-conservative SNPs in coding regions (Table S4), only 1 SNP (on the *Tdrd3* gene) predicted to be deleterious by PolyPhen and 8 genes had strong *cis*-eQTLs in whole brain, prefrontal cortex or liver expression datasets (Table S5). Of these potential gene candidates, *tudor domain containing 3* (*Tdrd3*) at 88.9 Mb on chromosome 14 had a *cis*-eQTL across multiple datasets and showed a very strong correlation (Pearson *r* = −0.79 in prefrontal cortex dataset) with plasma DOC levels across BXD strains (Figure S2).

None of the correlated behavioral phenotypes for ethanol or anxiety (Figures 3 and 4) showed a suggestive or significant QTL within the support intervals of the basal plasma or cerebral cortical DOC QTLs. However, pain sensitivity (Table S1) has a significant QTL on chromosome 4, similar to cerebral cortical DOC levels. Suggestive QTLs on the chromosome 4 support interval for basal cerebral cortical DOC levels are found for the kidney morphology trait (Wilms tumor 1 homolog negative cells per glomerular cross section in males, GN ID: 11020, Star et al., unpublished; Pearson *r* = 0.54, *p* = 0.007, *n* = 23) and for the photoreceptor density trait (GN ID: 10891, Guo et al., unpublished: Pearson *r* = −0.49, *p* = 0.003, *n* = 33, Table S1). No other traits were found to map in the support interval of the chromosome 14 QTL, associated with basal plasma DOC levels.

### Identification of genetic networks correlated with DOC levels

**Figure 6:**
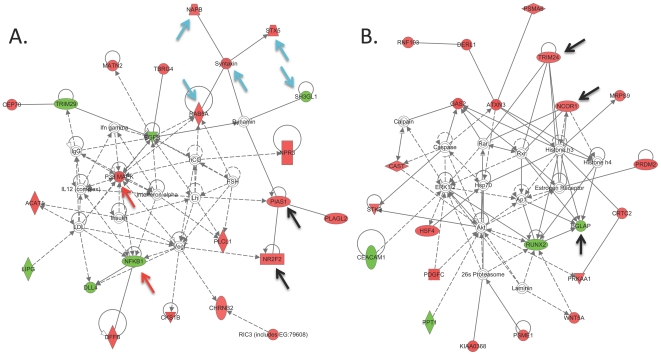
Genetic network analysis of brain gene expression correlating with basal cerebral cortical DOC levels across BXD strains. Genetic correlations (Pearson's *r*) between whole brain gene expression and basal cerebral cortical DOC levels were generated within GeneNetwork (www.genenetwork.org). A total of 458 genes (496 probesets) had *r* values of *p*<0.001 (Table S6). Gene networks were generated from this DOC gene set by Ingenuity Pathway Analysis. The top two scoring networks are portrayed. All colored gene symbols were contained within the DOC-correlated gene set with either positive (red) or negative (green) expression correlations with DOC across 20 BXD lines. Arrows indicate representative genes functioning in nuclear receptor action (black), inflammation (red), or vesicular trafficking and synaptic transmission (blue).

In light of the fact that DOC is known to regulate genes via nuclear receptors [31], we subsequently identified genes showing whole brain expression that correlated (Pearson's *r*) with basal cortical DOC levels across BXD strains, using existing microarray data within GeneNetwork (Table S6). A total of 458 genes showed significant expression correlations (*p*<0.001) with cortical DOC levels. Figure 6 shows the top two scoring gene networks obtained by Ingenuity Pathway analysis of this DOC-correlated gene set. Although varying cellular and biological functions were represented by genes in these genetic networks, the presence of multiple genes functioning in inflammation (Fig. 6A), vesicle trafficking (Fig. 6A) and nuclear receptor signaling (Fig. 6A and 6B) was particularly significant. We further determined if any of the 458 genes correlating with basal cerebral cortical DOC levels (Figure 6) are contained within the QTL for cerebral cortical DOC or show *trans*-linkage to cerebral cortical DOC levels. The only gene correlated with brain DOC levels and with *trans*-eQTL to the support interval of the chromosome 4 QTL is *Kiaa0368*, proteasome-associated protein ECM29 homolog (Pearson *r* = 0.76, *p*<0.001).

### Dexamethasone suppression of DOC in BXD strains

**Figure 7:**
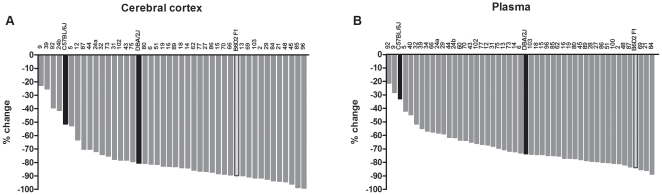
Variation in dexamethasone suppression of DOC levels across the BXD strains. Mice were injected with dexamethasone (0.075 mg/kg, sc) or saline at 8:00 am and were sacrificed 6 hours later. Data are expressed as % change of the average for dexamethasone-treated mice vs. the average for the respective saline-treated mice (*n* = 2–9/group/strain). The *x* axis reports the BXD strain number; C57BL/6J (B6), DBA/2J (D2) and B6D2 F1 hybrid are also indicated. Strains are plotted in order from the lowest to the highest suppression of DOC levels for cerebral cortex (42 BXD strains) or plasma (47 BXD strains), respectively.

**Figure 8:**
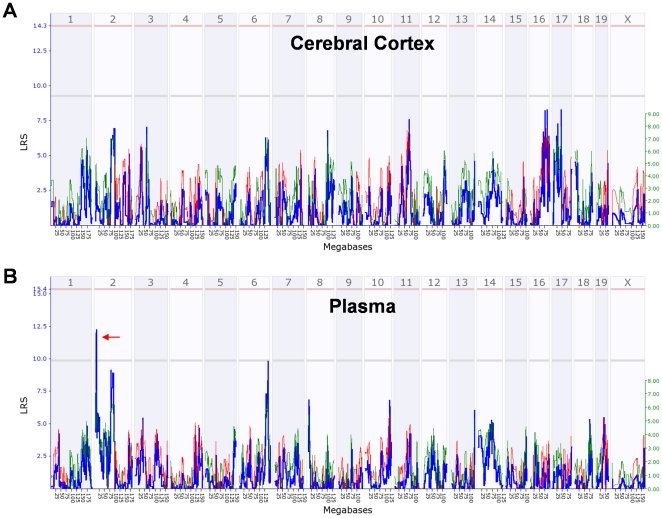
Genome-wide interval mapping for the dexamethasone-induced changes in (A) cerebral cortical and (B) plasma DOC levels across the BXD strains and their parental strains. Mice were injected with dexamethasone (0.075 mg/kg, sc) or saline at 8:00 am and were sacrificed 6 hours later. The % change of the average for dexamethasone-treated mice vs. the average for the respective saline-treated mice has been used for analysis. Panels show likelihood ratio statistic (LRS) scores across the entire genome. The y axis and the thick blue lines provide the LRS of the association between the trait and the genotypes of markers. The two horizontal lines are the suggestive (gray) and significance (red) thresholds computed using 1000 permutations. A suggestive QTL on chromosome 2 (red arrow) was identified. A positive additive coefficient (green line) indicates that D2 alleles increase trait values. A negative additive coefficient (red line) indicates that B6 alleles increase trait values.

Figure 7a shows the dexamethasone-induced changes in cerebral cortical DOC levels. Data is expressed as % change vs. the respective saline-treated strains. We observed a 4.4-fold variation in dexamethasone-induced changes in cerebral cortical DOC levels (range −22.6% to −99.1%); however, the majority of the strains showed a very strong suppression in DOC levels (−70 to −90%). Figure 7b shows the dexamethasone-induced changes in plasma DOC levels across the BXD strains. The pattern is very similar to cerebral cortex; we observed a 4.1-fold variation from −21.4% to −88.7%, with the majority of the strains showing greater than 70% suppression. In fact, there was a positive correlation between percent dexamethasone-induced changes in plasma vs. cerebral cortical DOC levels, across the strains so far examined (Pearson's *r* = 0.77, *p*<0.0001; Spearman's *r* = 0.65, *p*<0.0001, *n* = 45, data not shown). The interval mapping analysis identified a suggestive QTL on chromosome 2 for the dexamethasone-induced changes in plasma DOC levels (Figure 8b).

Variations in the dexamethasone-induced changes (%) in cerebral cortical and plasma DOC levels were also correlated with several phenotypes for ethanol sensitivity and anxiety, previously characterized in the BXD strains. Table S7 summarizes some of these significant correlations. Dexamethasone suppression of DOC levels was positively correlated with ethanol-induced ataxia, ethanol-induced hypothermia, ethanol-induced locomotor activity, and ethanol-induced anxiolysis. Dexamethasone suppression of DOC was inversely correlated with anxiety in some tests (light-dark box), but not others (elevated zero maze, locomotion in the center of open field arena). Furthermore, dexamethasone suppression of DOC was inversely correlated with volume and weight of several brain regions, as well as with neurogenesis in the rostral migratory stream.

We specifically searched for correlations between basal DOC levels or dexamethasone-induced changes in DOC levels and ethanol consumption, preference, ethanol-induced place preference or ethanol consumption following schedule-induced polydipsia. None of these correlations were found to be significant.

## Discussion

This study identifies the genetic regulation of basal and dexamethasone-induced levels of the neurosteroid DOC and links the genetic variation of this steroid with behavioral phenotypes and gene expression profiles previously determined across the BXD strains by multiple independent labs. In addition, we present evidence for a highly significant correlation between brain DOC and regulation of brain gene networks related to neuronal and nuclear receptor function.

To the best of our knowledge this is the first study to examine the genetic basis of variation in neurosteroid levels. Variation in basal levels of DOC resulted in heritability values of ∼0.37 and ∼0.32 even though basal levels did not significantly differ between the parental strains. Although the within-strain sample size is small for some of the strains (values range between 2 and 9), the total number of strains examined is greater than 40, thus allowing us enough power for QTL detection using recombinant inbred strains [32]. QTLs for basal DOC levels were identified in both cerebral cortex and plasma. The QTL for basal cerebral cortical DOC levels is located on chromosome 4 within a wide region comprised between 46 and 63 megabases. In contrast, the QTL for basal plasma DOC levels is located on chromosome 14 between 93 and 100 megabases; however, a suggestive QTL for basal plasma DOC levels was also identified on chromosome 4. It is perhaps somewhat surprising that there are no shared significant QTLs between plasma and cortical basal DOC levels, given the strong correlation (r = 0.78) between the two phenotypes. However, it is likely that the non-shared variance (∼40%) between the two traits is largely genetic and driving the two different QTLs. On the other hand, the involvement of different chromosomes, associated with basal DOC levels measured in the brain vs. the periphery, may reflect differential gene regulation of basal DOC biosynthesis in the cerebral cortex vs. adrenal. Indeed, adrenal glands are the major source of DOC production in the circulation. In agreement, a strong linkage between basal DOC levels and adrenal weight was observed for both cerebral cortex and plasma. Although these QTLs need to be confirmed and mapped at higher resolution by future studies, some of the candidate genes described in this analysis may provide novel information regarding the regulation of circulating DOC levels.

Many steroids, including DOC have a primary function as gene regulatory molecules by actions on nuclear receptors [31], [33]. In light of this we hypothesized that cortical DOC could be altering brain gene expression. We thus examined the correlation between cerebral cortical DOC levels and whole brain gene expression profiles across the BXD strains. Whole brain expression data was used as an exploratory screen since the possible regions of DOC action on brain gene expression are unclear. The networks portrayed in Figure 6 are suggestive of direct DOC effects on gene expression and support previous evidence from the literature for a role of neurosteroids in inflammation, synaptic transmission and neurotransmitter release [34], [35], [36], [37], [38]. Future studies will be required to validate the role of neurosteroids in regulation of these genetic networks.

Variation in basal levels of DOC was linked to several behavioral phenotypes previously determined in the BXD strains by independent labs. Among these phenotypes, the correlation between basal DOC levels and traits for ethanol sensitivity is of particular importance. Studies in rodents and humans have suggested that GABAergic neuroactive steroids may play a role in ethanol sensitivity. Systemic administration of ethanol increases brain and serum levels of DOC and the GABAergic neuroactive steroids in rodents [2], [3], . The ethanol-induced elevations of GABAergic neuroactive steroids contribute to several behavioral effects of ethanol in rodents, such as anticonvulsant effects [40], sedation [5], impairment of spatial memory [42], [43], anxiolytic-like [44] and antidepressant-like [45] actions. Each of these behavioral responses is prevented by pretreatment with the neurosteroid biosynthesis inhibitor finasteride and/or by prior adrenalectomy. Furthermore, administration of finasteride attenuates the subjective effects of ethanol in individuals homozygous for the A allele at the GABA_A_ receptor α2 subunit (GABRA2) gene polymorphism but not in individuals with the G allele (associated with alcohol dependence), suggesting a role for neuroactive steroids in mediating ethanol sensitivity in humans [46]. It has been hypothesized that GABAergic neuroactive steroids may protect against the risk for ethanol dependence [47], [48]. Diminished elevations of neuroactive steroids following ethanol exposure would result in reduced sensitivity to the anxiolytic, sedative, anticonvulsant, cognitive-impairing, and discriminative stimulus properties of ethanol [47]. Reduced sensitivity to ethanol is associated with greater risk for the development of alcoholism in individuals with genetic vulnerability to alcoholism [49], [50], [51], [52], [53]. The finding that those mouse strains with higher basal DOC levels also show greater ethanol sensitivity is consistent with this hypothesis. Higher basal DOC levels may reflect greater steroidogenesis in general or may result in higher production of its neuroactive metabolite THDOC. Studies are under way to examine this hypothesis.

Genetic linkage with behavioral phenotypes of ethanol sensitivity suggests overlap in the genes that control ethanol-induced sedation, ataxia, seizure susceptibility, locomotion, corticosterone levels and the regulation of basal DOC levels. It is important to note that variation in basal DOC levels was also linked to some anxiety phenotypes and seizure susceptibility. This is not surprising, given that systemic administration of neuroactive steroids induces anxiolytic and anticonvulsant properties [54], [55], [56] and neuroactive steroid levels are altered in several psychiatric disorders involving stress and anxiety [1], [57], [58].

Higher basal DOC levels were also linked to greater anxiety after restraint stress. Acute stress (like acute ethanol) activates the HPA axis and increases brain and circulating levels of GABAergic neuroactive steroids [1] as well as corticosterone, the major corticosteroid synthesized in rodents from DOC. GABAergic neuroactive steroids have anxiolytic properties when administered systemically [54], [55]. Thus, we might have predicted that those strains with higher basal DOC levels would have been less susceptible to anxiety, because of the protective role exerted by its neuroactive metabolite, THDOC. However, the heightened anxiety after restraint stress suggests that DOC is primarily metabolized to corticosterone. It should be noted that we found no correlation between basal DOC levels in our study and basal corticosterone levels measured 1 or 6 hours following saline injection [29]. This data is limited to the original panel of the BXD strains; thus, to better understand this relationship, studies of corticosterone levels in all the strains available, including the advanced panel, are warranted. Furthermore, the ratio of corticosterone to GABAergic metabolites after stress may provide more insight into the relationship between DOC levels and anxiety-like behavior.

Previous studies had suggested that dexamethasone suppression of DOC levels might correlate with ethanol consumption or preference, based on higher voluntary ethanol consumption in cynomolgus monkeys that exhibited weak suppression of DOC in response to dexamethasone [9]. In the monkey studies, ethanol consumption was measured across twelve months of voluntary consumption preceded by three months of scheduled induction of alcohol drinking, while drinking studies in the mice were measured across two to fifteen days with no induction procedure [27], [59], [60] (see also Matthews *et al.*, 2009, GN ID 11297; Lopez *et al.*, 2010, GN IDs: 12574–12580; Cook *et al.*, 2010, GN IDs 12565 and 12586, all unpublished on GeneNetwork). The fact that dexamethasone suppression of DOC levels was not predictive of ethanol consumption or preference across BXD strains is likely related to differences in the drinking paradigms. Alternatively, species differences may account for this discrepancy. Finally, it is likely that multiple genes located on different chromosomes may influence ethanol consumption and this may contribute to differential correlations between this trait and DOC suppression by dexamethasone.

This study focused on male mice only. Neurosteroid basal levels in female mice vary in relation to the estrus cycle phase [61]. Therefore, sex differences in basal DOC levels are likely to occur. Furthermore, sex differences and sex by strain interactions are not uncommon across studies of the BXD strains [62], [63], [64]. Future studies are needed to examine any potential sex differences in the genetic regulation of neurosteroid levels.

In conclusion, we have identified QTLs for basal levels of the neurosteroid DOC. Both basal DOC levels and dexamethasone suppression of DOC are positively correlated with ethanol sensitivity suggesting that the neurosteroid DOC could serve as a useful biomarker of alcohol phenotypes. Furthermore, DOC levels appear to be responsible for the regulation of networks of genes involved in the neuronal processes that underlie many aspects of brain function and likely the correlated ethanol phenotypes.

## Materials and Methods

### Animals

Male B6, D2 and B6D2 F1 hybrid mice (8 weeks old) were purchased from The Jackson Laboratory (Bar Harbor, ME, USA). BXD strains were either purchased from The Jackson Laboratory, or were acquired from the vivarium at the University of Tennessee Health Science Center (Memphis, TN, USA). After arrival at the animal facility, mice were allowed to acclimate for at least one week. They were housed four to six per cage under 12 h light, 12 h dark cycle (lights on from 0700 to 1900 h) and at a constant temperature of 22±2°C and relative humidity of 65%. They had free access to water and standard laboratory food at all times.

The BXD recombinant inbred strains have proved to be an extremely valuable reference population to study networks of phenotypes and their modulation by gene variants [18], [19], [20], [21]. The parental strains, B6 and D2, have been sequenced, and approximately two million SNPs between them have been identified. Most studies of BXD strains since 2001 have exploited ∼3,800 informative markers (mainly SNPs and microsatellites) selected from a set of ∼15,000 markers (see details in [20] and [65]). The full marker set can be downloaded as a text file at http://www.genenetwork.org/dbdoc/BXDGeno.html. The mean interval between informative markers is ∼0.7 Mb.

### Dexamethasone Administration

Mice were injected subcutaneously (sc) with dexamethasone (0.075 mg/kg) or saline at 8:00 am (lights on from 0700 to 1900 h) and were returned to their home-cage until sacrifice by decapitation 6 hours later. This protocol was adapted from previous work showing that administration of dexamethasone 0.1 mg/kg, sc, suppressed plasma corticosterone levels in B6 mice 6 hours after its administration [66]. The dose of dexamethasone was chosen because it induced a differential response in the parental strains [67]. To control for circadian fluctuations in DOC levels, the experiments were all performed at the same time of the day, as stated above. Furthermore, care was taken to minimize stress which would affect DOC levels. Following injections, all mice were left undisturbed in their home cage for 6 hours until sacrifice. Blood was collected from the trunk immediately after decapitation into lithium-heparin microtainer tubes (Becton Dickinson, Franklin Lakes, NJ, USA). It was centrifuged (1750 g for 15 min at 4°C) and serum samples were stored in plastic minivials at −80°C until use. The brain was rapidly extracted from the skull, dissected on ice, frozen on dry ice and stored at −80°C until DOC extraction and analysis. Animal care and handling throughout the experimental procedures followed National Institutes of Health Guidelines under University of North Carolina School of Medicine Institutional Animal Care and Use Committee approved protocols (Protocol 07-131). Adequate measures were taken to minimize pain or discomfort of the animals.

### DOC Radioimmunoassay

DOC was measured in mouse cerebral cortex and plasma by radioimmunoassay (RIA) as previously described [2], [9], with minor modifications. Individual cerebral cortexes were weighed, suspended in 3 ml of phosphate buffer and homogenized on ice with a sonic dismembrator. Homogenates were spiked with 1000 cpm of [^3^H]DOC (Specific activity = 50 Ci/mmol; American Radiolabeled Chemicals, Inc. Saint Louis, MO, USA) for recovery estimation. Samples were extracted three times in 4 ml aliquots of ethyl acetate. Plasma samples (100 µl) were extracted twice with 2 ml ethyl acetate/hexane (3∶2); 1000 cpm of [^3^H]DOC were added to each sample for recovery estimation. The dried extracts from cerebral cortex are resuspended in 2 ml RIA buffer, of which 0.5 ml is used for the assay (run in duplicate) and 0.5 ml is used for recovery determination. The dried extracts from plasma are resuspended in 1.5 ml RIA buffer, of which 0.5 ml is used for the assay (run in duplicate) and 0.3 ml is used for recovery determination. The DOC antiserum (MP Biomedicals, Solon, OH, USA) was diluted according to manufacturer's instructions. This antiserum is highly specific for DOC as shown by the following cross-reactivity tests: DOC 100%, 3α,5α-THDOC 4.7%, progesterone 2.5%, corticosterone 1.7%. Less than 1% cross-reactivity was observed for 3α,5α-THP, 3α-hydroxy-pregn-4-en-20-one, pregnenolone, 20-hydroxy-pregnen-4,3-one, testosterone, androstenedione, 17α-hydroxyprogesterone, 11-deoxycortisol, 5α-dihydrotestosterone, cortisol, cholesterol, 17β-estradiol, estrone, and estriol. Unknown samples were compared to concurrently run standards using a one-site competition model and adjusted for extraction efficiency. DOC values are expressed as ng/g of cerebral cortex or ng/ml of plasma. Intra-assay and inter-assay coefficients of variation were 5.6% and 13.5%, respectively for plasma and 6.9% and 12.2% for cerebral cortex.

### Statistical and bioinformatic analysis

ANOVAs were performed using a commercially available statistical program (GraphPad Prism 4.0, GraphPad Software, San Diego, CA, USA). Genetic data was analyzed using the statistical software available in GeneNetwork (www.genenetwork.org) and the R/QTL program within the R statistical framework. GeneNetwork allows for the analysis of networks of genes, transcripts and classic phenotype data sets [25]. Datasets for basal and dexamethasone-induced DOC levels were subjected to simple interval mapping analysis using Haley–Knott regression equations. Interval mapping was performed using the Haldane function, a 1 cM window, and marker maps for each chromosome that are very dense relative to recombination frequency in this cross. The thresholds for statistically significant (*p* value∼0.05) and suggestive (*p* value∼0.63) [68] genome-wide linkage were determined based on permutation tests [69]. Support intervals were calculated using R/QTL with a 97% Bayes credible interval [70]. Correlation analyses were performed using the log-transformed data in order to correct for non-normal distributions. Pearson's product moment and Spearman's rank correlations were computed using analytical tools integrated into GeneNetwork and using data sets of numerous BXD behavioral and physiological phenotypes, as well as array data of brain gene expression. Candidate genes for QTL support intervals were identified by mining the SNP database on GeneNetwork (http://genenetwork.org/webqtl/main.py?FormID=snpBrowser) for mis/non-sense polymorphisms between B6 and D2 strains within exons of genes within support intervals and by PolyPhen analysis (http://coot.embl.de/PolyPhen/). Additionally, BXD expression data from whole brain (UCHSC BXD Whole Brain M430 2.0 Nov06 RMA dataset), prefrontal cortex (VCU BXD PFC Sal M430 2.0 Dec06 RMA dataset), and liver (UNC Agilent G4121A Liver Males Only LOWESS Stanford Jan06 Dataset) were used to identify *cis*-acting expression QTL (*cis*-eQTL) within QTL support intervals using GeneNetwork. These *cis*-eQTLs show genetic linkage of their mRNA expression at a chromosomal site overlapping the gene location itself. In this case, it is genetically driven differences in expression of the candidate gene, rather than a genetic alteration in gene function per se, that is predicted to influence the quantitative trait (cerebral cortical DOC levels). We chose these datasets for mRNA expression correlation due to known effects of prefrontal cortex on HPA axis activity and drug addiction [71], [72], [73] and the role of the liver in steroid metabolism [74]. An adrenal BXD dataset was not currently available.

Molecular and genetic networks potentially regulated by cerebral cortex DOC were identified by correlating DOC levels with BXD expression data in GeneNetwork from whole brain (UCHSC BXD Whole Brain M430 2.0 Nov06 RMA dataset) using Pearson's product moment with a somewhat relaxed statistical filter (*p*<0.001) to enable network analysis. Pearson correlations were further analyzed for potential gene-gene network interactions using the Ingenuity Pathway Analysis bioinformatics platform. This platform superimposes gene set information on gene networks constructed from prior information from the biomedical literature, protein-protein interaction databases, known biochemical pathways and miRNA or transcription factor regulatory interactions.

## Supporting Information

Figure S1 Cluster maps to detect linkages for basal DOC levels in the cerebral cortex (A) and plasma (B). The demarcation along the long axis represents chromosomes 1 to X; red-yellow and blue-green color gradations code for intensity of linkage with higher trait values for D2 allele and B6 allele, respectively.
(TIFF)

Figure S2 The upper panel shows the interval map for *Tdrd3* in the prefrontal cortex (PFC) BXD saline dataset from GeneNetwork. This confirms a *cis*-eQTL at the position of the *Tdrd3* gene and the chromosome 14 QTL for basal plasma DOC. The lower panel shows correlation (Pearson's) of *Tdrd3* expression in PFC with plasma DOC levels.
(TIFF)

Table S1 Pearson's correlations of the log-transformed basal DOC data are reported. LORR: loss of righting reflex; HIC: handling-induced convulsions; VTA: ventral tegmental area; SN: substantia nigra.
(DOC)

Table S2 Genes containing nonsynonymous mutations between C57BL/6J (B6) and DBA/2J (D2) within the QTL support interval on chromosome 4.
(DOC)

Table S3 *Cis*-eQtTLs in the chromosome 4 support interval were identified using the GeneNetwork resources. The tissue for each database is indicated in bold. PFC: prefrontal cortex.
(DOC)

Table S4 Genes containing nonsynonymous mutations between C57BL/6J (B6) and DBA/2J (D2) within the QTL support interval on chromosome 14.
(DOC)

Table S5 *Cis*-eQtTLs in the chromosome 14 support interval were identified using the GeneNetwork resources. The tissue for each database is indicated in bold. PFC: prefrontal cortex.
(DOC)

Table S6 Gene expression correlations between basal cortical DOC and whole brain mRNA expression were done in GeneNetwork using the following parameters: Trait : BXDPublish : 12568, Database : UCHSC BXD Whole Brain M430 2.0 (Nov06) RMA, Citations: Please see http://132.192.47.32/reference.html.
(DOC)

Table S7 Pearson's correlations of the log-transformed DOC data are reported. HIC: handling-induced convulsions.
(DOC)
