# Correction: Genetic Analysis of the Neurosteroid Deoxycorticosterone and Its Relation to Alcohol Phenotypes: Identification of QTLs and Downstream Gene Regulation

**DOI:** 10.1371/annotation/ce7007ca-506f-4b54-b7ff-1e0a93d689f7

**Published:** 2011-06-30

**Authors:** Patrizia Porcu, Todd K. O'Buckley, Soomin C. Song, Jo Lynne Harenza, Lu Lu, Xusheng Wang, Robert W. Williams, Michael F. Miles, A. Leslie Morrow

One of the grant numbers cited in the funding information is incorrect. The complete and correct funding information is:

"This study was supported by the National Institute of Health NIAAA grants INIA-UO1-AA013641 (PP), INIA-UO1-AA016672 and R37-AA010564 (ALM), INIA-UO1-AA016662 (MFM), INIA-UO1-AA014425 (LL-RWW), INIA-UO1-AA13499, INIA-UO1-AA13513 and AA017590 (RWW) and by the University Research Council of the University of North Carolina at Chapel Hill (PP). The funders had no role in study design, data collection and analysis, decision to publish, or preparation of the manuscript."

