# Supplementary figures and images for: Genetic Analysis of the Neurosteroid Deoxycorticosterone and Its Relation to Alcohol Phenotypes: Identification of QTLs and Downstream Gene Regulation
Source: PLoS One. 2011 Apr 8;6(4):e18405. doi: 10.1371/journal.pone.0018405 (PMC3072994; doi:10.1371/journal.pone.0018405)

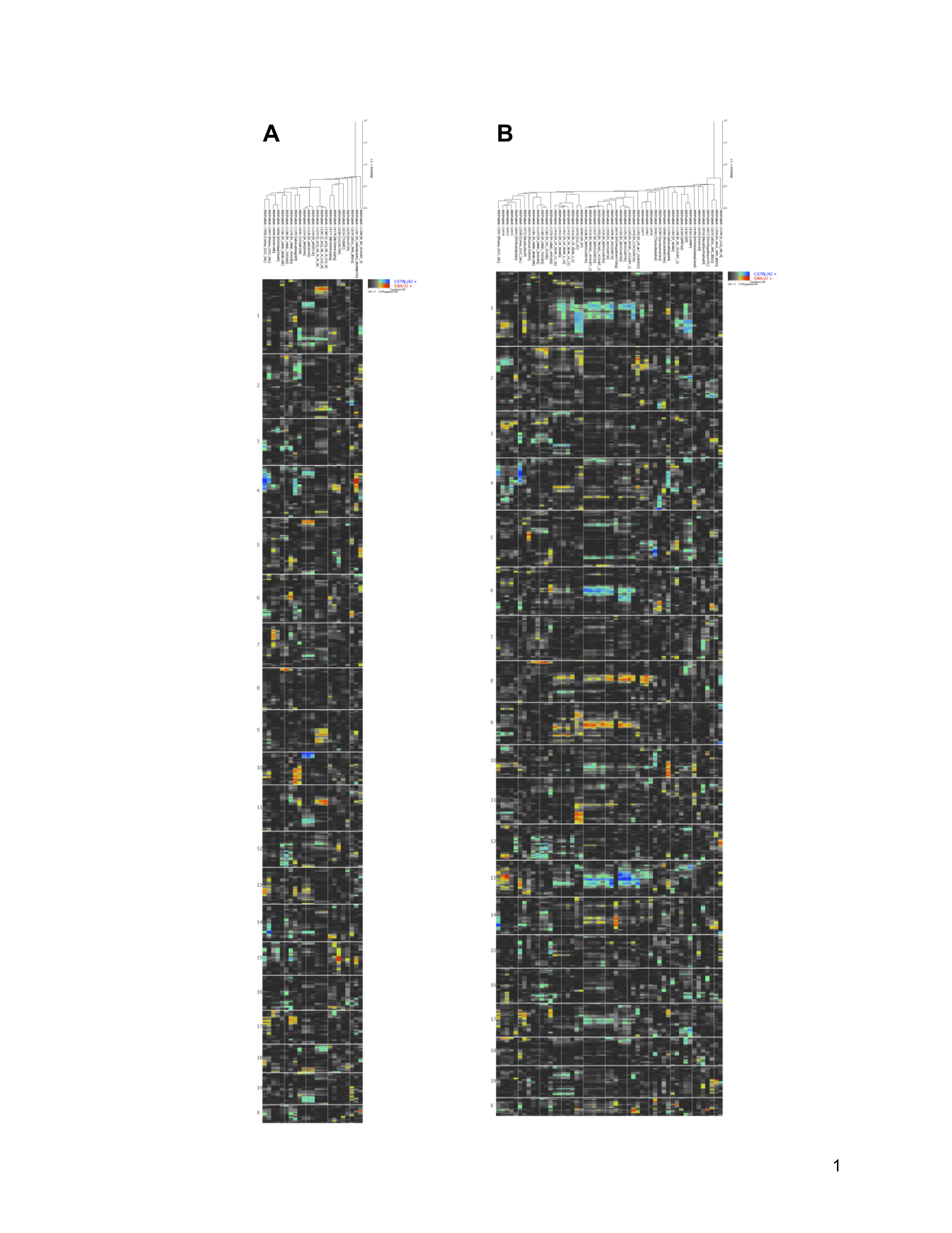

Supplement: Figure S1 — Cluster maps to detect linkages for basal DOC levels in the cerebral cortex (A) and plasma (B). The demarcation along the long axis represents chromosomes 1 to X; red-yellow and blue-green color gradations code for intensity of linkage with higher trait values for D2 allele and B6 allele, respectively. (TIFF) [file pone.0018405.s001.tif]

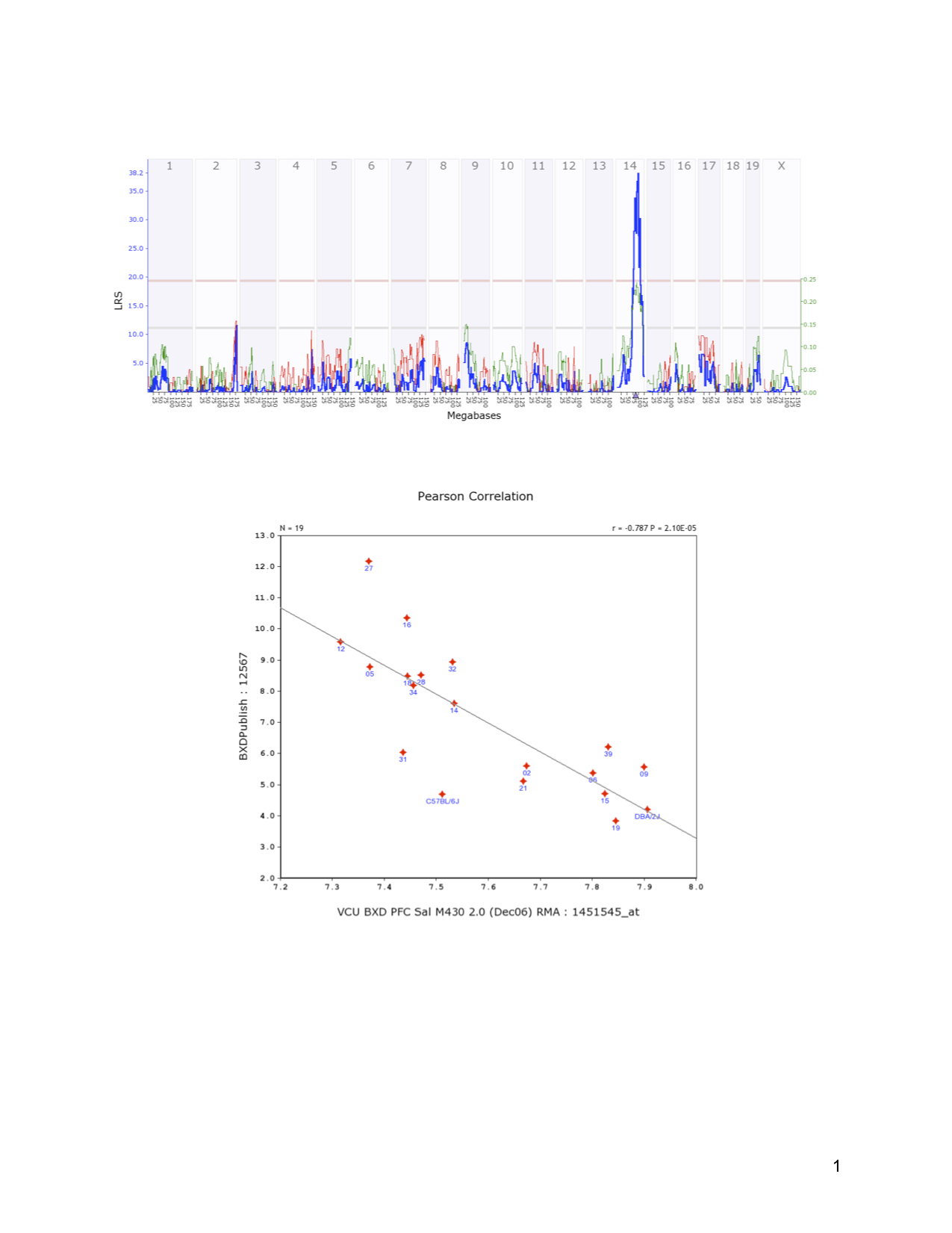

Supplement: Figure S2 — The upper panel shows the interval map for Tdrd3 in the prefrontal cortex (PFC) BXD saline dataset from GeneNetwork. This confirms a cis-eQTL at the position of the Tdrd3 gene and the chromosome 14 QTL for basal plasma DOC. The lower panel shows correlation (Pearson's) of Tdrd3 expression in PFC with plasma DOC levels. (TIFF) [file pone.0018405.s002.tif]
