# Supplementary material for: Genetic Analysis of the Neurosteroid Deoxycorticosterone and Its Relation to Alcohol Phenotypes: Identification of QTLs and Downstream Gene Regulation
Source: PLoS One. 2011 Apr 8;6(4):e18405. doi: 10.1371/journal.pone.0018405 (PMC3072994; doi:10.1371/journal.pone.0018405)
Supplement: Table S1 — Pearson's correlations of the log-transformed basal DOC data are reported. LORR: loss of righting reflex; HIC: handling-induced convulsions; VTA: ventral tegmental area; SN: substantia nigra. (DOC) [file pone.0018405.s003.doc]

**Table S1.** Genetic correlations between basal cerebral cortical or plasma DOC levels and behavioral phenotypes across the BXD strains.

|  |  |  | **Cerebral cortical DOC** | | | **Plasma DOC** | | |
| --- | --- | --- | --- | --- | --- | --- | --- | --- |
| **Phenotypes** | **Reference** | **GN ID** | **r** | **p** | **n** | **r** | **p** | **n** |
|  |  |  |  |  |  |  |  |  |
| Blood ethanol concentrations 60 min after ethanol 2 g/kg, ip | (Grisel et al. 2002) | 10177 | -0.63 | 0.003 | 19 |  |  |  |
| Blood ethanol concentrations 60 min after ethanol 3 g/kg, ip | (Grisel et al. 2002) | 10178 | -0.58 | 0.008 | 19 |  |  |  |
| Ethanol-induced sedation: LORR after 4.1 g/kg, ip, males | (Rodriguez et al. 1994) | 10586 | 0.63 | 0.008 | 16 |  |  |  |
| Ethanol withdrawal-induced seizures: HIC 4 hrs after 4 g/kg, ip, males | (Philip et al. 2010) | 11378 | 0.54 | 0.009 | 22 | 0.52 | 0.01 | 23 |
| Ethanol withdrawal-induced seizures: HIC 6 hrs after 4 g/kg, ip, males | (Philip et al. 2010) | 11379 |  |  |  | 0.47 | 0.023 | 23 |
| Ethanol withdrawal-induced seizures: HIC 7 hrs after 4 g/kg, ip, males | (Philip et al. 2010) | 11380 | 0.46 | 0.029 | 22 | 0.43 | 0.038 | 23 |
| Ethanol withdrawal-induced seizures: HIC after 4 g/kg, ip, males | (Philip et al. 2010) | 11382 |  |  |  | 0.47 | 0.048 | 18 |
| Ethanol-induced locomotion: distance traveled 0-20 min - 2.25 g/kg, ip, males | (Philip et al. 2010) | 11448 | 0.33 | 0.047 | 36 |  |  |  |
| Ethanol-induced locomotion, 2 g/kg, ip, males | (Cunningham 1995) | 10088 | -0.50 | 0.049 | 16 |  |  |  |
| Ethanol-induced ataxia, 2.5 g/kg, ip | (Browman and Crabbe 2000) | 10042 | 0.46 | 0.049 | 19 |  |  |  |
| Ethanol-induced ataxia, 1.75 mg/kg ip, males | (Kirstein et al. 2002) | 10350 |  |  |  | 0.49 | 0.024 | 21 |
| Ethanol-induced plasma corticosterone levels 1 hr after 4 g/kg ip | (Roberts et al. 1995) | 10573 |  |  |  | 0.67 | 0.003 | 17 |
| Ethanol-induced plasma corticosterone levels 6 hrs after 4 g/kg ip, males | (Roberts et al. 1995) | 10580 |  |  |  | 0.53 | 0.033 | 16 |
| Ethanol-induced anxiolysis: elevated zero maze, 1.8 g/kg, last 5 min, males | (Cook et al, unpublished) | 12383 |  |  |  | -0.31 | 0.037 | 44 |
| Tolerance to ethanol-induced hypothermia, 2 g/kg | (Crabbe et al. 1994) | 10070 |  |  |  | -0.56 | 0.018 | 17 |
| Tolerance to ethanol-induced hypothermia, 4 g/kg | (Crabbe et al. 1994) | 10072 |  |  |  | -0.52 | 0.033 | 17 |
| Chronic ethanol induced seizures: HIC 15 hrs after 1.5 g/kg, ip challenge | (Crabbe et al. 1983) | 10081 | -0.80 | 0.029 | 7 |  |  |  |
|  |  |  |  |  |  |  |  |  |
| Anxiety: time in open arms of elevated plus maze | (Brigman et al. 2009) | 11012 | -0.53 | 0.042 | 15 |  |  |  |
| Open field behavior, vertical activity rears, 0-5 min, males | (Philip et al. 2010) | 11414 |  |  |  | -0.33 | 0.034 | 41 |
| Open field behavior, vertical activity rears, 0-20 min, males | (Philip et al. 2010) | 11413 |  |  |  | -0.43 | 0.004 | 41 |
| Open field behavior, vertical activity rears, 5-10 min, males | (Philip et al. 2010) | 11417 |  |  |  | -0.36 | 0.019 | 41 |
| Open field behavior, vertical activity rears, 10-15 min, males | (Philip et al. 2010) | 11415 |  |  |  | -0.44 | 0.003 | 41 |
| Open field behavior, vertical activity rears, 15-20 min, males | (Philip et al. 2010) | 11416 |  |  |  | -0.47 | 0.002 | 41 |
| Open field behavior, vertical activity rears, 30-45 min, males | (Philip et al. 2010) | 11351 |  |  |  | -0.36 | 0.018 | 41 |
| Open field behavior, vertical activity rears 45-60 min, males | (Philip et al. 2010) | 11352 |  |  |  | -0.38 | 0.014 | 41 |
| Open field behavior, vertical activity rears in the periphery 0-60 min, males | (Philip et al. 2010) | 11533 |  |  |  | -0.39 | 0.01 | 42 |
| Open field behavior, vertical activity rears in the periphery 15-30 min, males | (Philip et al. 2010) | 11527 |  |  |  | -0.35 | 0.021 | 42 |
| Open field behavior, vertical activity rears in the periphery 30-45 min, males | (Philip et al. 2010) | 11528 |  |  |  | -0.42 | 0.005 | 42 |
| Open field behavior, vertical activity rears in the periphery 45-60 min, males | (Philip et al. 2010) | 11529 |  |  |  | -0.38 | 0.013 | 42 |
| Open field behavior, vertical activity rears in the center 0-60 min, males | (Philip et al. 2010) | 11355 |  |  |  | -0.35 | 0.024 | 41 |
| Open field behavior, vertical activity rears in the center 30-45 min, males | (Philip et al. 2010) | 11516 |  |  |  | -0.31 | 0.048 | 42 |
| Fear conditioning response, activity in altered context, males | (Philip et al. 2010) | 11392 |  |  |  | -0.44 | 0.004 | 41 |
| Restraint stress: light-dark transitions after saline, 10 min test | Putman and Miles, unpublished | 10978 |  |  |  | -0.62 | 0.004 | 19 |
| Restraint stress: light-dark transitions after saline, 5 min test | Putman and Miles, unpublished | 10960 |  |  |  | -0.56 | 0.01 | 19 |
| Restraint stress: activity in closed quadrants of elevated zero maze, 10 min, males | Cook et al, unpublished | 12474 |  |  |  | 0.39 | 0.008 | 44 |
| Restraint stress: activity in closed quadrants of elevated zero maze, last 5 min, males | Cook et al, unpublished | 12473 |  |  |  | 0.31 | 0.038 | 44 |
| Restraint stress: time in open quadrants of elevated zero maze, last 5 min, males | Cook et al, unpublished | 12467 |  |  |  | -0.37 | 0.01 | 44 |
| Acoustic startle response, % response, males | (Philip et al. 2010) | 11429 |  |  |  | 0.32 | 0.046 | 40 |
| Acoustic startle response, prepulse inhibition at 70db, males | (Philip et al. 2010) | 11426 |  |  |  | -0.32 | 0.046 | 40 |
|  |  |  |  |  |  |  |  |  |
| Naloxone-induced morphine withdrawal: vertical activity, 0-15 min, males | (Philip et al. 2010) | 11358 | -0.40 | 0.014 | 36 | -0.43 | 0.005 | 41 |
| Naloxone-induced morphine withdrawal: wet dog shakes, males | (Philip et al. 2010) | 11362 |  |  |  | -0.44 | 0.004 | 41 |
| Morphine-induced locomotion: open field behavior, 0-15 min, males | (Philip et al. 2010) | 11341 | 0.33 | 0.048 | 36 |  |  |  |
| Morphine-induced locomotion: open field behavior, 45-60 min, males | (Philip et al. 2010) | 11348 |  |  |  | -0.32 | 0.039 | 41 |
| Morphine metabolism | (Wahlstrom et al. 1986) | 10693 |  |  |  | 0.86 | 0.004 | 8 |
|  |  |  |  |  |  |  |  |  |
| Cocaine (5 mg/kg) open field activity, males | (Jones et al. 1999) | 10289 | -0.55 | 0.027 | 16 |  |  |  |
| Cocaine (15 mg/kg, ip) exploratory activity | (Jones et al. 1999) | 10301 | 0.54 | 0.028 | 16 |  |  |  |
| Cocaine open field activity, vertical rears, 0-60 min, males | (Philip et al. 2010) | 11555 |  |  |  | -0.35 | 0.021 | 42 |
| Cocaine (10 mg/kg, ip) open field activity, vertical rears, 15-30 min, males | (Philip et al. 2010) | 11478 |  |  |  | -0.32 | 0.036 | 42 |
| Cocaine (10 mg/kg, ip) open field activity, vertical rears, 30-45 min, males | (Philip et al. 2010) | 11479 |  |  |  | -0.31 | 0.048 | 42 |
|  |  |  |  |  |  |  |  |  |
| Seizure susceptibility: baseline handling-induced convulsions, males | (Philip et al. 2010) | 11381 | 0.60 | 0.002 | 22 | 0.60 | 0.002 | 23 |
| Seizure susceptibility to high atmospheric pressure | (Plomin et al. 1991) | 10507 |  |  |  | -0.54 | 0.030 | 16 |
| Seizure threshold pressure at compression rate of 1000 atm/hr | (McCall and Jr. 1981) | 10388 |  |  |  | -0.53 | 0.035 | 16 |
|  |  |  |  |  |  |  |  |  |
| Adrenal total weight, males | Di Curzio and Goldowitz, 2011 | 11299 | 0.52 | 0.0005 | 39 | 0.52 | 0.0002 | 44 |
| Adrenal zona fasciculata width, males | Di Curzio and Goldowitz, 2011 | 11270 |  |  |  | 0.33 | 0.026 | 44 |
| Pain response, Hargreaves’ test thermal nociception, males | (Philip et al. 2010) | 11307 | -0.35 | 0.038 | 35 | -0.33 | 0.037 | 40 |
| Morris water maze: swim speed during acquisition of task | (Kempermann and Gage 2002) | 10814 | -0.74 | 0.032 | 8 |  |  |  |
| Motor performance: improvement in rotarod training | (Brigman et al. 2009) | 11005 | 0.52 | 0.044 | 15 |  |  |  |
| Raffinose bitter taste response (% consumption) | (Lush 1986) | 10382 | 0.62 | 0.041 | 11 |  |  |  |
| Saccharin sweet taste response (% consumption) | (Lush 1989) | 10384 |  |  |  | -0.59 | 0.025 | 14 |
| Acesulfame sweet taste response (% consumption) | (Lush 1989) | 10385 |  |  |  | -0.58 | 0.029 | 14 |
| Tyrosine hydroxylase neurons in substantia nigra compacta, males | (Hitzemann et al. 2003) | 10224 | -0.60 | 0.006 | 19 |  |  |  |
| Tyrosine hydroxylase neurons in ventral tegmental area, males | (Hitzemann et al. 2003) | 10223 | -0.47 | 0.041 | 19 |  |  |  |
| Photoreceptor density (rods and cones) | Guo et al, unpublished | 10891 | -0.49 | 0.003 | 33 |  |  |  |
| Dopamine receptor 1 (DRD1) protein density in prefrontal cortex, males | (Jones et al. 1999) | 10255 | -0.67 | 0.015 | 12 | -0.63 | 0.027 | 12 |
| Dopamine receptor 1 (DRD1) protein density in VTA + SN, males | (Jones et al. 1999) | 10258 | -0.63 | 0.025 | 12 |  |  |  |
| Dopamine transporter SLC6A3 protein density in prefrontal cortex, males | (Jones et al. 1999) | 10279 | -0.59 | 0.043 | 12 |  |  |  |
| Zinc levels in nucleus accumbens, males | (Jones et al. 1999) | 10722 |  |  |  | 0.68 | 0.014 | 12 |
| Neurogenesis, BrdU-labeled cells in the rostral migratory stream, males | (Philip et al. 2010) | 11556 |  |  |  | -0.41 | 0.031 | 27 |

**References**

Brigman JL, Mathur P, Lu L, Williams RW, Holmes A (2009) Genetic relationship between anxiety-related and fear-related behaviors in BXD recombinant inbred mice. Behav Pharmacol 20: 204-9

Browman KE, Crabbe JC (2000) Quantitative trait loci affecting ethanol sensitivity in BXD recombinant inbred mice. Alcohol Clin Exp Res 24: 17-23

Crabbe JC, Belknap JK, Mitchell SR, Crawshaw LI (1994) Quantitative trait loci mapping of genes that influence the sensitivity and tolerance to ethanol-induced hypothermia in BXD recombinant inbred mice. J Pharmacol Exp Ther 269: 184-192

Crabbe JC, Kosobud A, Young ER, Janowsky JS (1983) Polygenic and single-gene determination of responses to ethanol in BXD/Ty recombinant inbred mouse strains. Neurobehav Toxicol Teratol 5: 181-7

Cunningham CL (1995) Localization of genes influencing ethanol-induced conditioned place preference and locomotor activity in BXD recombinant inbred mice. Psychopharmacology 120: 28-41

Di Curzio DL, Goldowitz D (2011) The genetic basis of adrenal gland weight and structure in BXD recombinant inbred mice. Mamm Genome, in press.

Grisel JE, Metten P, Wenger CD, Merrill CM, Crabbe JC (2002) Mapping of quantitative trait loci underlying ethanol metabolism in BXD recombinant inbred mouse strains. Alcohol Clin Exp Res 26: 610-6

Hitzemann R, Hitzemann B, Rivera S, Gatley J, Thanos P, Shou LL, Williams RW (2003) Dopamine D2 receptor binding, Drd2 expression and the number of dopamine neurons in the BXD recombinant inbred series: genetic relationships to alcohol and other drug associated phenotypes. Alcohol Clin Exp Res 27: 1-11

Jones BC, Tarantino LM, Rodriguez LA, Reed CL, McClearn GE, Plomin R, Erwin VG (1999) Quantitative-trait loci analysis of cocaine-related behaviours and neurochemistry. Pharmacogenetics 9: 607-17

Kempermann G, Gage FH (2002) Genetic determinants of adult hippocampal neurogenesis correlate with acquisition, but not probe trial performance, in the water maze task. Eur J Neurosci 16: 129-36

Kirstein SL, Davidson KL, Ehringer MA, Sikela JM, Erwin VG, Tabakoff B (2002) Quantitative trait loci affecting initial sensitivity and acute functional tolerance to ethanol-induced ataxia and brain cAMP signaling in BXD recombinant inbred mice. J Pharmacol Exp Ther 302: 1238-45

Lush IE (1986) The genetics of tasting in mice. IV. The acetates of raffinose, galactose and beta-lactose. Genet Res 47: 117-23

Lush IE (1989) The genetics of tasting in mice. VI. Saccharin, acesulfame, dulcin and sucrose. Genet Res 53: 95-9

McCall RD, Jr. FD (1981) Evidence that two loci predominantly determine the difference in susceptibility to the high pressure neurologic syndrome type I seizure in mice. Genetics 99: 285-307

Philip VM, Duvvuru S, Gomero B, Ansah TA, Blaha CD, Cook MN, Hamre KM, Lariviere WR, Matthews DB, Mittleman G, Goldowitz D, Chesler EJ (2010) High-throughput behavioral phenotyping in the expanded panel of BXD recombinant inbred strains. Genes Brain Behav 9: 129-159

Plomin R, McClearn GE, Gora-Maslak G, Neiderhiser JM (1991) Use of recombinant inbred strains to detect quantitative trait loci associated with behavior. Behav Genet 21: 99-116

Roberts AJ, Phillips TJ, Belknap JK, Finn DA, Keith LD (1995) Genetic analysis of the corticosterone response to ethanol in BXD recombinant inbred mice. Behav Neurosci 109: 1199-208

Rodriguez LA, Plomin R, Blizard DA, Jones BC, McClearn GE (1994) Alcohol acceptance, preference, and sensitivity in mice. I. Quantitative genetic analysis using BXD recombinant inbred strains. Alcohol Clin Exp Res 18: 1416-22

Wahlstrom A, Hammar L, Lundin LG, Rane A (1986) Morphine metabolism in mouse brain. NIDA Res Monogr 75: 603-6
