# Supplementary material for: Genetic Analysis of the Neurosteroid Deoxycorticosterone and Its Relation to Alcohol Phenotypes: Identification of QTLs and Downstream Gene Regulation
Source: PLoS One. 2011 Apr 8;6(4):e18405. doi: 10.1371/journal.pone.0018405 (PMC3072994; doi:10.1371/journal.pone.0018405)
Supplement: Table S2 — Genes containing nonsynonymous mutations between C57BL/6J (B6) and DBA/2J (D2) within the QTL support interval on chromosome 4. (DOC) [file pone.0018405.s004.doc]

**Table S2.** Genes containing nonsynonymous mutations between C57BL/6J (B6) and DBA/2J (D2) within the QTL support interval on chromosome 4.

| **Gene** | **Domain** | **Position** | **SNPs (B6/D2)** | **Amino acid change** | **PolyPhen prediction** |
| --- | --- | --- | --- | --- | --- |
| *Grin3a* | exon8 | 49682924 | A/G | C/R |  |
| *Cylc2* | exon5 | 51242340 | A/G | E/G |  |
| *Smc2* | exon8 | 52463836 | G/T | V/L |  |
| *Smc2* | exon10 | 52470210 | G/A | S/N |  |
| *Smc2* | exon11 | 52471380 | G/A | A/T |  |
| *Nipsnap3b* | exon1 | 53024873 | G/T | R/L | deleterious |
| *Nipsnap3b* | exon2 | 53027923 | A/G | N/D |  |
| *Nipsnap3b* | exon2 | 53027934 | C/A | F/L |  |
| *Zfp462* | exon3 | 55021767 | C/T | A/V |  |
| *Zfp462* | exon3 | 55021826 | G/T | A/S |  |
| *Zfp462* | exon3 | 55022589 | C/A | P/Q |  |
| *Zfp462* | exon3 | 55023179 | G/A | A/T |  |
| *Zfp462* | exon3 | 55025173 | A/G | I/M |  |
| *Zfp462* | exon3 | 55025705 | C/T | P/S |  |
| *Zfp462* | exon3 | 55026375 | T/C | V/A | deleterious |
| *Zfp462* | exon13 | 55093539 | T/G | S/A |  |
| *Zfp462* | exon13 | 55093708 | A/G | K/R |  |
| *Rad23b* | exon4 | 55383139 | G/A | A/T |  |
| *Rad23b* | exon4 | 55383199 | C/G | P/A |  |
| *Rad23b* | exon4 | 55383226 | G/A | A/T |  |
| *Rad23b* | exon9 | 55398345 | A/G | S/G |  |
| *Ikbkap* | exon31 | 56775142 | C/T | V/I |  |
| *Ikbkap* | exon18 | 56789591 | A/C | V/G |  |
| *Ikbkap* | exon11 | 56800766 | T/C | I/V |  |
| *D730040F13Rik* | exon6 | 56936317 | T/C | H/R | deleterious |
| *Epb4.1l4b* | exon13 | 57083943 | C/T | V/I |  |
| *Olfr267* | exon1 | 58797695 | T/C | M/V |  |
| *AI314180* | exon40 | 58824582 | A/T | D/E |  |
| *Zkscan16* | exon2 | 58959006 | G/C | G/A |  |
| *Zkscan16* | exon2 | 58959126 | A/G | D/G | deleterious |
| *Zkscan16* | exon2 | 58959157 | G/C | W/C | deleterious |
| *Zkscan16* | exon2 | 58959360 | C/G | T/S | deleterious |
| *Ptgr1* | exon10 | 58979614 | A/G | Y/H |  |
| *Ptgr1* | exon10 | 58979622 | C/T | C/Y |  |
| *Susd1* | exon10 | 59378740 | G/A | A/V | deleterious |
| *Slc46a2* | exon1 | 59926974 | C/G | G/R | deleterious |
| *Mup10* | exon1 | 60594951 | T/G | M/L |  |
