# Supplementary material for: Genetic Analysis of the Neurosteroid Deoxycorticosterone and Its Relation to Alcohol Phenotypes: Identification of QTLs and Downstream Gene Regulation
Source: PLoS One. 2011 Apr 8;6(4):e18405. doi: 10.1371/journal.pone.0018405 (PMC3072994; doi:10.1371/journal.pone.0018405)
Supplement: Table S3 — Cis-eQtTLs in the chromosome 4 support interval were identified using the GeneNetwork resources. The tissue for each database is indicated in bold. PFC: prefrontal cortex. (DOC) [file pone.0018405.s005.doc]

**Table S3.** *Cis*-eQTLs within the chromosome 4 support interval.

| **DataBase** | **ProbeSet ID / Record ID** | **Symbol** | **Description** | **Probe Target** | **Gene ID** | **Mb** | **Locus at Peak** | **Marker at peak** | **Max LRS** | **P value** |
| --- | --- | --- | --- | --- | --- | --- | --- | --- | --- | --- |
| UCHSC BXD **Whole Brain** M430 2.0 (Nov06) RMA | 1441142_at | *2700081L22Rik* | RIKEN cDNA 2700081L22 gene |  | 72648 | 52.4432 | 49.7924 | rs13477695 | 39.096 | 0 |
| VCU BXD **PFC** Sal M430 2.0 (Dec06) RMA | 1441142_at | *2700081L22Rik* | RIKEN cDNA 2700081L22 gene |  | 72648 | 52.4432 | 49.7924 | rs13477695 | 57.044 | 0.000021 |
| UCHSC BXD **Whole Brain** M430 2.0 (Nov06) RMA | 1436185_at | *AI314180* | expressed sequence AI314180 | far 3' UTR | 230249 | 58.8118 | 55.0688 | rs13477715 | 22.929 | 0.0022 |
| UCHSC BXD **Whole Brain** M430 2.0 (Nov06) RMA | 1435407_at | *AU067697* | expressed sequence AU067697 | putative exon (from ESTs) | 100346 | 58.9749 | 55.0688 | rs13477715 | 19.627 | 0.026 |
| UNC Agilent G4121A **Liver** LOWESS Stanford (Jan06) Males | A_51_P374077 | *AW547365* | expressed sequence AW547365 |  | 100434 | 53.6352 | 49.7924 | rs13477695 | 27.062 | 0.00067 |
| UNC Agilent G4121A **Liver** LOWESS Stanford (Jan06) Males | A_51_P251410 | *Epb4.1l4b* | erythrocyte protein band 4.1-like 4b |  | 54357 | 57.0749 | 61.3443 | rs13477735 | 25.783 | 0.00077 |
| UCHSC BXD **Whole Brain** M430 2.0 (Nov06) RMA | 1456570_at | *EPB41L4B* | erythrocyte membrane protein band 4.1 like 4B | far 3' UTR |  | 57.0079 | 55.0688 | rs13477715 | 43.188 | 0 |
| VCU BXD **PFC** Sal M430 2.0 (Dec06) RMA | 1456570_at | *EPB41L4B* | erythrocyte membrane protein band 4.1 like 4B | far 3' UTR |  | 57.0079 | 55.0688 | rs13477715 | 57.877 | 0.000006 |
| UNC Agilent G4121A **Liver** LOWESS Stanford (Jan06) Males | A_51_P217047 | *Gng10* | guanine nucleotide binding protein (G protein), gamma 10 |  | 14700 | 59.0542 | 56.5912 | rs6239799 | 27.140 | 0.00069 |
| UNC Agilent G4121A **Liver** LOWESS Stanford (Jan06) Males | A_51_P114878 | *Ikbkap* | inhibitor of kappa light polypeptide enhancer in B-cells, kinase complex-associated protein |  | 230233 | 56.7640 | 56.5912 | rs6239799 | 21.902 | 0.011 |
| UCHSC BXD **Whole Brain** M430 2.0 (Nov06) RMA | 1448967_at | *Nipsnap3a* | nipsnap homolog 3A | mid to distal 3' UTR | 66536 | 53.0344 | 55.0688 | rs13477715 | 16.028 | 0.012 |
| UCHSC BXD **Whole Brain** M430 2.0 (Nov06) RMA | 1424222_s_at | *Rad23b* | RAD23b homolog | mid 3' UTR | 19359 | 55.4031 | 55.0688 | rs13477715 | 29.005 | 0.00025 |
| UCHSC BXD **Whole Brain** M430 2.0 (Nov06) RMA | 1450903_at | *Rad23b* | RAD23b homolog | 3' UTR | 19359 | 55.4036 | 55.0688 | rs13477715 | 16.262 | 0.04 |
| UCHSC BXD **Whole Brain** M430 2.0 (Nov06) RMA | 1455420_at | *Rad23b* | RAD23b homolog (S. cerevisiae) |  | 19359 | 55.4046 | 55.0688 | rs13477715 | 34.347 | 0.00011 |
| VCU BXD **PFC** Sal M430 2.0 (Dec06) RMA | 1424222_s_at | *Rad23b* | RAD23b homolog | mid 3' UTR | 19359 | 55.4031 | 55.0688 | rs13477715 | 49.898 | 0.000014 |
| VCU BXD **PFC** Sal M430 2.0 (Dec06) RMA | 1450903_at | *Rad23b* | RAD23b homolog | 3' UTR | 19359 | 55.4036 | 55.0688 | rs13477715 | 15.391 | 0.018 |
| VCU BXD **PFC** Sal M430 2.0 (Dec06) RMA | 1437120_at | *Snx30* | sorting nexin family member 30 | proximal 3' UTR | 209131 | 59.9120 | 55.0688 | rs13477715 | 29.891 | 0.000045 |
