# Supplementary material for: Genetic Analysis of the Neurosteroid Deoxycorticosterone and Its Relation to Alcohol Phenotypes: Identification of QTLs and Downstream Gene Regulation
Source: PLoS One. 2011 Apr 8;6(4):e18405. doi: 10.1371/journal.pone.0018405 (PMC3072994; doi:10.1371/journal.pone.0018405)
Supplement: Table S4 — Genes containing nonsynonymous mutations between C57BL/6J (B6) and DBA/2J (D2) within the QTL support interval on chromosome 14. (DOC) [file pone.0018405.s006.doc]

**Table S4.** Genes containing nonsynonymous mutations between C57BL/6J (B6) and DBA/2J (D2) within the QTL support interval on chromosome 14.

| **Gene** | **Domain** | **Position** | **SNPs (B6/D2)** | **Amino acid change** | **PolyPhen prediction** |
| --- | --- | --- | --- | --- | --- |
| *Diap3* | exon28 | 87056221 | T/C | K/R |  |
| *Diap3* | exon24 | 87228885 | T/C | I/V |  |
| *Diap3* | exon16 | 87366380 | C/A | G/C |  |
| *Diap3* | exon11 | 87385469 | C/T | S/N |  |
| *Tdrd3* | exon11 | 87905647 | C/G | T/S |  |
| *Tdrd3* | exon11 | 87905830 | A/G | K/R | deleterious |
| *Tdrd3* | exon11 | 87905889 | C/T | L/F |  |
| *Tdrd3* | exon11 | 87906060 | T/G | C/G |  |
| *Tdrd3* | exon11 | 87906099 | G/T | V/F |  |
| *Tdrd3* | exon11 | 87906152 | G/T | R/S |  |
| *Pcdh20* | exon3 | 88866853 | C/G | L/F |  |
| *Pcdh20* | exon2 | 88870769 | A/G | L/S |  |
| *Pcdh9* | exon1 | 94287183 | C/T | V/I |  |
| *4921530L21Rik* | exon2 | 96281370 | T/C | Y/H |  |
| *4921530L21Rik* | exon2 | 96281829 | A/G | K/E |  |
