# Supplementary material for: Genetic Analysis of the Neurosteroid Deoxycorticosterone and Its Relation to Alcohol Phenotypes: Identification of QTLs and Downstream Gene Regulation
Source: PLoS One. 2011 Apr 8;6(4):e18405. doi: 10.1371/journal.pone.0018405 (PMC3072994; doi:10.1371/journal.pone.0018405)
Supplement: Table S5 — Cis-eQtTLs in the chromosome 14 support interval were identified using the GeneNetwork resources. The tissue for each database is indicated in bold. PFC: prefrontal cortex. (DOC) [file pone.0018405.s007.doc]

**Table S5.** *Cis*-eQTLs within the chromosome 14 support interval.

| **DataBase** | **ProbeSet ID / Record ID** | **Symbol** | **Description** | **Probe Target** | **Gene ID** | **Mb** | **Locus at Peak** | **Marker at peak** | **Max LRS** | **P value** |
| --- | --- | --- | --- | --- | --- | --- | --- | --- | --- | --- |
| UCHSC BXD **Whole Brain** M430 2.0 (Nov06) RMA | 1457973_at | *2310043D08Rik* | ESTs | Intronic |  | 94.2269 | 96.5609 | gnf14.093.344 | 31.229 | 0.00053 |
| VCU BXD **PFC** Sal M430 2.0 (Dec06) RMA | 1457973_at | *2310043D08Rik* | ESTs | Intronic |  | 94.2269 | 96.5609 | gnf14.093.344 | 84.406 | 0.000005 |
| UCHSC BXD **Whole Brain** M430 2.0 (Nov06) RMA | 1434664_at | *2410129H14Rik* | RIKEN cDNA 2410129H14 | mid 3' UTR | 76789 | 99.4347 | 88.3589 | UT_9_81.82101 | 22.288 | 0.0075 |
| VCU BXD **PFC** Sal M430 2.0 (Dec06) RMA | 1434664_at | *2410129H14Rik* | RIKEN cDNA 2410129H14 | mid 3' UTR | 76789 | 99.4347 | 96.5609 | gnf14.093.344 | 36.719 | 0.000065 |
| UCHSC BXD **Whole Brain** M430 2.0 (Nov06) RMA | 1429478_at | *6720463M24Rik* | RIKEN cDNA 6720463M24 gene |  | 77744 | 99.4466 | 96.5609 | gnf14.093.344 | 21.177 | 0.014 |
| VCU BXD **PFC** Sal M430 2.0 (Dec06) RMA | 1429478_at | *6720463M24Rik* | RIKEN cDNA 6720463M24 gene |  | 77744 | 99.4466 | 96.5609 | gnf14.093.344 | 41.178 | 0.000006 |
| UCHSC BXD **Whole Brain** M430 2.0 (Nov06) RMA | 1444724_at | *BC023488* | cDNA sequence BC023488 | Intronic or AK137405 | 237221 | 94.1225 | 88.3589 | UT_9_81.82101 | 38.005 | 0.000093 |
| VCU BXD **PFC** Sal M430 2.0 (Dec06) RMA | 1444724_at | *BC023488* | cDNA sequence BC023488 | Intronic or AK137405 | 237221 | 94.1225 | 98.6592 | rs3692362 | 21.505 | 0.0043 |
| VCU BXD **PFC** Sal M430 2.0 (Dec06) RMA | 1436920_at | *C030033F14Rik* | RIKEN cDNA C030033F14 gene |  | 382924 | 84.9334 | 82.4486 | rs13482276 | 65.964 | 0.000003 |
| UNC Agilent G4121A **Liver** LOWESS Stanford (Jan06) Males | A_51_P131744 | *C030033F14Rik* | RIKEN cDNA C030033F14 gene |  | 77386 | 84.9368 | 82.4486 | rs13482276 | 44.946 | 0 |
| UCHSC BXD **Whole Brain** M430 2.0 (Nov06) RMA | 1436465_at | *Klhl1* | kelch-like 1 (Drosophila) |  | 93688 | 96.5046 | 96.5609 | gnf14.093.344 | 45.474 | 0.000043 |
| VCU BXD **PFC** Sal M430 2.0 (Dec06) RMA | 1442659_at | *Pcdh9* | hypothetical protein A730003J17 |  | 211712 | 94.2854 | 96.5609 | gnf14.093.344 | 27.302 | 0.002 |
| VCU BXD **PFC** Sal M430 2.0 (Dec06) RMA | 1458269_at | *Pcdh9* | hypothetical protein A730003J17 |  | 211712 | 94.2874 | 92.5943 | rs13482313 | 25.038 | 0.0022 |
| UCHSC BXD **Whole Brain** M430 2.0 (Nov06) RMA | 1451545_at | *Tdrd3* | tudor domain containing 3 |  | 219249 | 87.9063 | 82.4486 | rs13482276 | 34.905 | 0.00025 |
| VCU BXD **PFC** Sal M430 2.0 (Dec06) RMA | 1451545_at | *Tdrd3* | tudor domain containing 3 |  | 219249 | 87.9063 | 96.5609 | gnf14.093.344 | 38.203 | 0.000078 |
