# Supplementary material for: Genetic Analysis of the Neurosteroid Deoxycorticosterone and Its Relation to Alcohol Phenotypes: Identification of QTLs and Downstream Gene Regulation
Source: PLoS One. 2011 Apr 8;6(4):e18405. doi: 10.1371/journal.pone.0018405 (PMC3072994; doi:10.1371/journal.pone.0018405)
Supplement: Table S6 — Gene expression correlations between basal cortical DOC and whole brain mRNA expression were done in GeneNetwork using the following parameters: Trait : BXDPublish : 12568, Database : UCHSC BXD Whole Brain M430 2.0 (Nov06) RMA, Citations: Please see http://132.192.47.32/reference.html. (DOC) [file pone.0018405.s008.doc]

**Table S6.** Brain gene expression correlations with basal cortical DOC across BXD strains.

| **Record ID** | **Entrez Gene ID** | **Symbol** | **Chr** | **Megabase** | **Mean Expr** | **Sample r** | **Sample p(r)** |
| --- | --- | --- | --- | --- | --- | --- | --- |
| 1418167_at | 83383 | Tcfap4 | 16 | 4.54 | 7.28 | 0.906 | 5.49E-10 |
| 1448720_at | 69928 | Lrrc40 | 3 | 157.73 | 8.53 | 0.882 | 1.18E-08 |
| 1435092_at | 11861 | Arl4a | 12 | 40.76 | 7.85 | 0.852 | 1.87E-07 |
| 1422241_a_at | 54405 | Ndufa1 | X | 34.73 | 13.30 | -0.845 | 3.20E-07 |
| 1418472_at | 11484 | Aspa | 11 | 73.12 | 9.73 | 0.844 | 3.54E-07 |
| 1445671_at |  | Zfp59 | 17 | 55.79 | 5.48 | 0.836 | 6.44E-07 |
| 1440798_x_at | 54562 | Lrrc6 | 15 | 66.21 | 5.61 | 0.830 | 9.63E-07 |
| 1428385_at | 71779 | March8 | 6 | 116.36 | 9.09 | 0.828 | 1.13E-06 |
| 1455091_at | 235542 | Ppp2r3a | 9 | 101.01 | 7.70 | 0.827 | 1.16E-06 |
| 1455117_at | 215112 | BC062185 | 10 | 53.26 | 6.47 | 0.821 | 1.73E-06 |
| 1456204_at | 66487 | Snhg8 | 14 | 31.94 | 7.88 | 0.815 | 2.49E-06 |
| 1430530_s_at | 67824 | Nmral1 | 16 | 4.71 | 7.33 | 0.815 | 2.55E-06 |
| 1448654_at | 56428 | Mtch2 | 2 | 90.71 | 9.87 | 0.814 | 2.60E-06 |
| 1452669_at | 66306 | 2810012G03Rik | 18 | 34.93 | 10.77 | -0.813 | 2.87E-06 |
| 1437313_x_at | 19886 | Ros1 | 10 | 25.83 | 5.88 | 0.803 | 5.01E-06 |
| 1439516_at | 18148 | Npm1 | 11 | 33.05 | 8.22 | 0.793 | 8.61E-06 |
| 1450998_at | 65020 | Zfp110 | 7 | 13.44 | 6.32 | 0.792 | 9.10E-06 |
| 1450112_a_at | 14453 | Gas2 | 7 | 59.25 | 7.30 | 0.790 | 1.02E-05 |
| 1416698_a_at | 54124 | Cks1b | 3 | 89.22 | 7.83 | 0.789 | 1.04E-05 |
| 1423284_at | 67729 | Mansc1 | 6 | 134.56 | 8.04 | 0.789 | 1.07E-05 |
| 1419123_a_at | 54635 | Pdgfc | 3 | 81.02 | 7.21 | 0.788 | 1.10E-05 |
| 1424075_at | 68115 | 9430016H08Rik | 1 | 57.47 | 8.02 | 0.786 | 1.20E-05 |
| 1450494_x_at | 26367 | Ceacam2 | 7 | 26.25 | 8.44 | -0.786 | 1.21E-05 |
| 1424603_at | 58911 | Sumf1 | 6 | 108.06 | 6.87 | 0.785 | 1.30E-05 |
| 1444437_at | 17847 | Usp34 | 11 | 23.36 | 6.77 | 0.783 | 1.42E-05 |
| 1429468_at | 56692 | Map2k1ip1 | 3 | 137.58 | 9.83 | 0.782 | 1.49E-05 |
| 1435858_at | 320500 | A930001M12Rik | 4 | 40.42 | 5.24 | 0.781 | 1.58E-05 |
| 1455255_at | 76425 | 2310003C23Rik | 2 | 180.46 | 9.13 | 0.780 | 1.65E-05 |
| 1444673_at | 23857 | Dmtf1 | 5 | 9.16 | 5.43 | 0.780 | 1.65E-05 |
| 1443500_at | 17354 | Mllt10 | 2 | 17.99 | 8.58 | 0.779 | 1.70E-05 |
| 1459305_at | 328365 | Rai17 | 14 | 26.40 | 9.41 | -0.778 | 1.82E-05 |
| 1442044_at |  | BM242890 | 4 | 86.50 | 5.61 | 0.775 | 2.08E-05 |
| 1429642_at | 67492 | Anubl1 | 6 | 116.28 | 6.89 | 0.774 | 2.12E-05 |
| 1459615_at |  | B130052P14Rik | 14 | 28.25 | 4.52 | 0.774 | 2.14E-05 |
| 1418720_at | 26895 | Cops7b | 1 | 88.50 | 9.41 | -0.774 | 2.17E-05 |
| 1434408_at | 110616 | Atxn3 | 12 | 103.16 | 8.06 | 0.774 | 2.18E-05 |
| 1437492_at | 210719 | Mkx | 18 | 6.94 | 6.64 | 0.773 | 2.22E-05 |
| 1435236_at | 245695 | A630018P17Rik | X | 162.94 | 6.07 | 0.773 | 2.28E-05 |
| 1457182_at | 102209 | Snapc2 | 8 | 4.25 | 5.63 | 0.772 | 2.38E-05 |
| 1416653_at | 20912 | Stxbp3a | 3 | 108.60 | 8.54 | 0.772 | 2.41E-05 |
| 1424306_at | 83603 | Elovl4 | 9 | 83.67 | 9.82 | 0.768 | 2.83E-05 |
| 1443232_at | 545859 | Vax2os2 | 6 | 83.64 | 8.97 | -0.768 | 2.83E-05 |
| 1434691_at | 72193 | Sfrs2ip | 15 | 96.24 | 8.88 | 0.767 | 2.91E-05 |
| 1434094_at | 230678 | 6330530A05Rik | 4 | 118.21 | 8.77 | 0.767 | 2.98E-05 |
| 1417473_a_at | 106564 | 6330579B17Rik | 4 | 119.09 | 5.43 | 0.766 | 3.09E-05 |
| 1449283_a_at | 29857 | Mapk12 | 15 | 88.96 | 8.84 | 0.765 | 3.19E-05 |
| 1433563_s_at | 67819 | Derl1 | 15 | 57.70 | 9.10 | 0.762 | 3.69E-05 |
| 1446145_at | 230861 | Eif4g3 | 4 | 137.67 | 9.59 | -0.762 | 3.74E-05 |
| 1442371_at | 76908 | 1110048D14Rik | 5 | 29.56 | 7.55 | 0.760 | 3.96E-05 |
| 1435186_s_at | 71710 | 1200008A14Rik | 3 | 14.57 | 8.30 | 0.760 | 4.01E-05 |
| 1439734_at |  | Mmp15 | 8 | 97.90 | 8.93 | -0.760 | 4.06E-05 |
| 1436185_at | 230249 | AI314180 | 4 | 58.81 | 8.66 | 0.759 | 4.09E-05 |
| 1433022_at | 70185 | 2310068G24Rik | 12 | 82.35 | 8.12 | -0.759 | 4.11E-05 |
| 1455460_at | 320521 | 6430547I21Rik | 3 | 17.70 | 9.59 | 0.759 | 4.12E-05 |
| 1432500_at | 382770 | C330014B19Rik | 13 | 66.60 | 5.34 | -0.757 | 4.49E-05 |
| 1425776_a_at | 232196 | C87436 | 6 | 86.42 | 7.90 | 0.756 | 4.71E-05 |
| 1424182_at | 110446 | Acat1 | 9 | 53.39 | 8.92 | 0.755 | 4.92E-05 |
| 1440656_at | 94253 | E130207I19Rik | 13 | 14.41 | 9.11 | -0.752 | 5.58E-05 |
| 1434991_at | 109082 | 1110064L07Rik | 13 | 50.53 | 7.97 | 0.749 | 6.26E-05 |
| 1453918_at | 320924 | Ccbe1 | 18 | 66.43 | 5.29 | -0.749 | 6.33E-05 |
| 1455621_at | 240066 | BC066107 | 17 | 33.02 | 5.11 | 0.747 | 6.76E-05 |
| 1427956_at | 67711 | Nspc1 | 6 | 22.68 | 7.77 | 0.747 | 6.76E-05 |
| 1450417_a_at | 67427 | Rps20 | 4 | 3.76 | 5.12 | 0.746 | 7.11E-05 |
| 1447801_x_at | 26409 | Map3k7 | 4 | 32.11 | 4.39 | 0.746 | 7.12E-05 |
| 1440115_at |  | A130096K20 | 2 | 28.06 | 6.64 | -0.746 | 7.15E-05 |
| 1436528_at | 107250 | Kazald1 | 19 | 45.15 | 7.70 | 0.744 | 7.49E-05 |
| 1429068_at | 72842 | 2810488G03Rik | 6 | 8.46 | 6.20 | 0.744 | 7.61E-05 |
| 1449913_at | 22678 | Zfp2 | 11 | 50.71 | 7.07 | 0.744 | 7.67E-05 |
| 1447844_at |  | D930030O05Rik | 17 | 27.34 | 5.66 | 0.744 | 7.73E-05 |
| 1458355_x_at | 103784 | Monad | 11 | 17.13 | 5.83 | 0.743 | 8.00E-05 |
| 1456574_at | 57867 | AA407452 | 6 | 28.19 | 5.89 | 0.743 | 8.04E-05 |
| 1455087_at | 52480 | D7Ertd715e | 7 | 67.11 | 7.24 | 0.741 | 8.46E-05 |
| 1458679_a_at | 69694 | Tatdn1 | 15 | 58.74 | 8.34 | 0.741 | 8.52E-05 |
| 1438278_a_at | 80744 | BC003993 | 2 | 83.80 | 7.08 | 0.741 | 8.54E-05 |
| 1457090_at | 74490 | 5430432N15Rik | 8 | 27.26 | 6.60 | 0.741 | 8.73E-05 |
| 1438593_at | 18139 | Zfml | 6 | 83.87 | 5.16 | 0.740 | 8.78E-05 |
| 1451023_at | 15168 | Hcn3 | 3 | 88.95 | 9.67 | -0.740 | 8.80E-05 |
| 1432871_at | 74389 | 4932429P19Rik | 12 | 33.11 | 8.31 | -0.739 | 9.29E-05 |
| 1450921_at | 66408 | Aptx | 4 | 40.64 | 7.88 | 0.739 | 9.35E-05 |
| 1439495_at | 71101 | 4933407H18Rik | 5 | 33.76 | 7.24 | 0.738 | 9.43E-05 |
| 1421075_s_at | 13123 | Cyp7b1 | 3 | 17.97 | 8.35 | 0.738 | 9.56E-05 |
| 1435184_at | 319900 | B430320C24Rik | 15 | 11.77 | 7.22 | 0.738 | 9.57E-05 |
| 1428515_at | 69747 | 2410012H22Rik | 11 | 62.08 | 8.43 | 0.737 | 9.80E-05 |
| 1444661_at | 233919 | Gpr26 | 7 | 139.18 | 9.06 | -0.737 | 9.87E-05 |
| 1438727_at | 242418 | Wdr32 | 4 | 45.39 | 7.10 | 0.737 | 1.01E-04 |
| 1437051_at | 13368 | Dffb | 4 | 153.34 | 7.01 | 0.737 | 1.01E-04 |
| 1454376_at |  | 4833410I11Rik | Un | 0.00 | 5.73 | -0.736 | 1.02E-04 |
| 1428114_at | 108052 | Slc14a1 | 18 | 78.30 | 7.68 | 0.736 | 1.02E-04 |
| 1449673_s_at | 233871 | Atxn2l | 7 | 133.63 | 5.82 | -0.736 | 1.02E-04 |
| 1436277_at | 57295 | Icmt | 4 | 151.68 | 7.61 | 0.736 | 1.04E-04 |
| 1433655_at | 67150 | Rnf141 | 7 | 117.96 | 10.48 | 0.735 | 1.08E-04 |
| 1440221_at | 53382 | Txnl1 | 18 | 63.82 | 7.44 | 0.735 | 1.09E-04 |
| 1439035_at | 218100 | Zfp322a | 13 | 23.45 | 9.76 | -0.734 | 1.12E-04 |
| 1426494_at | 69934 | Rg9mtd3 | 4 | 45.32 | 8.59 | 0.732 | 1.19E-04 |
| 1451166_a_at | 75565 | Ccdc101 | 7 | 133.82 | 7.30 | 0.732 | 1.19E-04 |
| 1444112_at | 22724 | Zbtb7b | 3 | 89.20 | 7.55 | 0.732 | 1.20E-04 |
| 1454658_at | 216136 | Ilvbl | 10 | 78.05 | 9.62 | 0.732 | 1.21E-04 |
| 1453156_s_at | 77219 | Zadh1 | 12 | 85.66 | 7.92 | 0.731 | 1.22E-04 |
| 1434607_at | 78394 | Ddx52 | 11 | 83.77 | 7.57 | 0.731 | 1.23E-04 |
| 1442042_at |  | 2810021J22Rik | 15 | 82.74 | 8.65 | -0.731 | 1.23E-04 |
| 1436411_at | 268878 | Atp13a5 | 16 | 29.23 | 8.18 | 0.731 | 1.25E-04 |
| 1454822_x_at | 494504 | Apcdd1 | 18 | 63.11 | 7.94 | 0.730 | 1.27E-04 |
| 1453431_at | 72495 | 2610206C17Rik | 7 | 91.92 | 7.06 | -0.730 | 1.28E-04 |
| 1444450_at | 242681 | 9530096D07Rik | 4 | 131.86 | 6.85 | 0.730 | 1.28E-04 |
| 1417765_a_at | 11722 | Amy1 | 3 | 113.26 | 8.30 | 0.729 | 1.32E-04 |
| 1437351_at | 319478 | Cxxc4 | 3 | 133.92 | 9.94 | 0.729 | 1.32E-04 |
| 1434869_at | 219249 | Tdrd3 | 14 | 87.91 | 6.00 | 0.729 | 1.33E-04 |
| 1459659_at | 72465 | 6330575P09Rik | Un | 0.00 | 6.64 | 0.729 | 1.35E-04 |
| 1426034_a_at | 12393 | Runx2 | 17 | 44.74 | 7.48 | -0.729 | 1.35E-04 |
| 1459191_at | 74053 | Grip1 | 10 | 118.94 | 7.60 | -0.728 | 1.40E-04 |
| 1432747_at |  | 4933403J19Rik | 19 | 44.00 | 5.97 | -0.727 | 1.42E-04 |
| 1437075_at | 242506 | Frmd3 | 4 | 73.83 | 5.57 | 0.727 | 1.43E-04 |
| 1417782_at | 67260 | Lass4 | 8 | 4.53 | 7.11 | 0.727 | 1.44E-04 |
| 1457563_at | 13649 | Egfr | 11 | 16.66 | 4.83 | -0.727 | 1.45E-04 |
| 1436146_at | 7493 | 4930481A15Rik | 19 | 5.42 | 5.31 | -0.727 | 1.45E-04 |
| 1443401_at | 69807 | Trim32 | 4 | 65.28 | 8.08 | 0.726 | 1.48E-04 |
| 1443069_at | 18750 | Prkca | 11 | 108.04 | 8.44 | -0.726 | 1.48E-04 |
| 1431806_at | 77059 | 4931408D14Rik | 19 | 37.33 | 8.12 | -0.726 | 1.49E-04 |
| 1459882_at | 66403 | Asf1a | 10 | 53.33 | 4.73 | 0.725 | 1.52E-04 |
| 1420708_at | 22036 | Traip | 9 | 107.87 | 8.95 | -0.724 | 1.58E-04 |
| 1435504_at | 78785 | Rsnl2 | 17 | 72.21 | 9.61 | 0.724 | 1.59E-04 |
| 1416607_at | 70984 | 4931406C07Rik | 9 | 15.09 | 9.14 | 0.724 | 1.59E-04 |
| 1416907_at | 22099 | Tsn | 1 | 120.20 | 10.04 | 0.724 | 1.61E-04 |
| 1443696_s_at | 226243 | Habp2 | 19 | 56.38 | 7.69 | -0.723 | 1.63E-04 |
| 1448267_at | 56389 | Stx5a | 19 | 8.83 | 7.89 | 0.723 | 1.63E-04 |
| 1455118_at | 382117 | D9Ertd402e | 9 | 122.74 | 9.53 | 0.723 | 1.64E-04 |
| 1434160_at | 233410 | Zfp592 | 7 | 88.19 | 9.61 | -0.723 | 1.65E-04 |
| 1437364_at | 230027 | Coq3 | 4 | 21.84 | 7.28 | 0.723 | 1.66E-04 |
| 1445723_at | 227120 | Plcl1 | 1 | 55.81 | 8.47 | 0.722 | 1.68E-04 |
| 1460348_at | 71890 | Mad2l2 | 4 | 147.52 | 7.75 | 0.722 | 1.70E-04 |
| 1454252_at | 77209 | 8030453O22Rik | 6 | 39.48 | 7.48 | -0.722 | 1.70E-04 |
| 1425786_a_at | 26386 | Hsf4 | 8 | 107.80 | 7.46 | 0.722 | 1.72E-04 |
| 1443794_x_at | 100608 | Noc4l | 5 | 111.08 | 7.16 | 0.721 | 1.75E-04 |
| 1441964_at | 66145 | 1110003F05Rik | 17 | 10.40 | 7.99 | 0.720 | 1.80E-04 |
| 1421375_a_at | 20200 | S100a6 | 3 | 90.42 | 8.29 | 0.720 | 1.82E-04 |
| 1446681_at | 12647 | Chat | 14 | 33.22 | 7.10 | 0.720 | 1.82E-04 |
| 1424465_at | 19107 | Dnajc3 | 14 | 119.35 | 7.24 | 0.720 | 1.84E-04 |
| 1436636_at |  | A230078I05Rik | 17 | 46.99 | 7.71 | 0.719 | 1.86E-04 |
| 1433291_at |  | C030045M22Rik | 12 | 39.23 | 7.03 | -0.719 | 1.88E-04 |
| 1452411_at | 214345 | Lrrc1 | 9 | 77.28 | 7.60 | 0.719 | 1.89E-04 |
| 1426913_at | 16987 | Lss | 10 | 76.02 | 7.98 | 0.719 | 1.89E-04 |
| 1435719_at | 50793 | AI448984 | 4 | 34.53 | 8.88 | 0.719 | 1.90E-04 |
| 1448104_at | 104776 | Aldh6a1 | 12 | 85.77 | 9.77 | 0.719 | 1.92E-04 |
| 1429251_at | 110593 | Prdm2 | 4 | 142.72 | 8.64 | 0.718 | 1.94E-04 |
| 1424370_s_at | 228769 | Psmf1 | 2 | 151.54 | 7.98 | 0.718 | 1.97E-04 |
| 1439091_at | 211651 | Fancd2 | 6 | 113.55 | 8.00 | -0.718 | 1.97E-04 |
| 1429419_at | 66353 | 2310007A19Rik | 3 | 94.27 | 6.46 | 0.717 | 2.02E-04 |
| 1417941_at | 67311 | Hdhd4 | 2 | 150.86 | 10.16 | 0.717 | 2.04E-04 |
| 1458723_at |  | BG066801 | 11 | 60.45 | 6.57 | -0.716 | 2.07E-04 |
| 1451566_at | 235050 | BC005471 | 9 | 22.08 | 8.57 | 0.716 | 2.10E-04 |
| 1437276_at | 102423 | Hinfp | 9 | 44.10 | 6.55 | 0.716 | 2.11E-04 |
| 1460391_at | 67059 | Ola1 | 2 | 72.93 | 7.90 | 0.715 | 2.13E-04 |
| 1448745_s_at | 16939 | Lor | 3 | 91.88 | 7.14 | 0.715 | 2.13E-04 |
| 1422159_at | 19023 | Ppef2 | 5 | 92.66 | 5.57 | -0.715 | 2.14E-04 |
| 1433975_at | 234854 | Cdk10 | 8 | 125.76 | 9.47 | 0.715 | 2.15E-04 |
| 1434604_at | 226982 | Eif5b | 1 | 38.11 | 9.15 | 0.715 | 2.15E-04 |
| 1456653_a_at | 270685 | Mthfd1l | 10 | 6.19 | 8.66 | 0.714 | 2.23E-04 |
| 1440809_at | 56872 | Swap70 | 7 | 117.41 | 8.19 | -0.714 | 2.24E-04 |
| 1438079_at | 237775 | BC050078 | 11 | 59.28 | 5.67 | 0.714 | 2.24E-04 |
| 1441550_at |  | 9330184L24Rik | X | 117.05 | 5.16 | 0.714 | 2.25E-04 |
| 1452992_at | 66440 | Cdc26 | 4 | 93.47 | 7.61 | 0.713 | 2.27E-04 |
| 1436791_at | 22418 | Wnt5a | 14 | 29.34 | 7.62 | 0.713 | 2.31E-04 |
| 1433762_at | 21885 | Tle1 | 4 | 71.86 | 7.08 | 0.713 | 2.33E-04 |
| 1451085_at | 223665 | C030006K11Rik | 15 | 76.55 | 7.90 | 0.713 | 2.34E-04 |
| 1417239_at | 12626 | Cetn3 | 13 | 81.93 | 9.72 | 0.712 | 2.39E-04 |
| 1427007_at | 74131 | 1200013B08Rik | X | 45.51 | 7.91 | -0.711 | 2.44E-04 |
| 1422653_at | 68121 | C030018L16Rik | 9 | 99.20 | 7.93 | 0.711 | 2.44E-04 |
| 1424611_x_at | 227682 | Trub2 | 2 | 29.63 | 7.76 | 0.711 | 2.47E-04 |
| 1420801_at | 18142 | Npas1 | 7 | 17.04 | 7.33 | 0.711 | 2.49E-04 |
| 1420016_at | 19063 | Ppt1 | 4 | 122.53 | 5.59 | -0.710 | 2.51E-04 |
| 1415830_at | 26429 | Orc5l | 5 | 21.99 | 8.52 | 0.710 | 2.51E-04 |
| 1447190_at |  | 2010205A11Rik | 2 | 154.46 | 9.10 | -0.710 | 2.54E-04 |
| 1440202_at | 72139 | 2610044O15Rik | 13 | 74.74 | 6.84 | 0.710 | 2.54E-04 |
| 1421074_at | 13123 | Cyp7b1 | 3 | 17.97 | 8.48 | 0.710 | 2.55E-04 |
| 1435275_at | 333182 | Cox6b2 | 7 | 4.70 | 7.65 | 0.710 | 2.58E-04 |
| 1454865_at | 77031 | Slc9a8 | 2 | 167.30 | 8.48 | 0.709 | 2.66E-04 |
| 1417517_at | 54711 | Plagl2 | 2 | 153.05 | 7.50 | 0.708 | 2.69E-04 |
| 1449082_at | 50530 | Mfap5 | 6 | 122.48 | 7.59 | -0.708 | 2.70E-04 |
| 1448645_at | 17692 | Msl31 | X | 165.09 | 9.16 | 0.708 | 2.70E-04 |
| 1440262_at |  | AI854107 | 9 | 27.16 | 9.85 | -0.708 | 2.74E-04 |
| 1416426_at | 271457 | Rab5a | 17 | 53.65 | 11.92 | 0.707 | 2.81E-04 |
| 1428780_at | 71776 | 1300017K07Rik | 11 | 117.73 | 7.04 | 0.707 | 2.83E-04 |
| 1429137_at | 69962 | 2810422O20Rik | 1 | 165.93 | 7.85 | 0.707 | 2.84E-04 |
| 1448969_at | 68017 | Ftsj2 | 5 | 140.80 | 7.99 | 0.706 | 2.87E-04 |
| 1429109_at | 77853 | Msl2 | 9 | 101.01 | 10.51 | -0.706 | 2.93E-04 |
| 1452960_at | 240880 | Scyl3 | 1 | 165.88 | 8.76 | 0.706 | 2.93E-04 |
| 1437495_at | 270669 | Mbtps2 | X | 154.00 | 5.96 | 0.705 | 2.94E-04 |
| 1444563_at |  | AW494124 | 2 | 167.17 | 9.07 | -0.705 | 2.94E-04 |
| 1419442_at | 17181 | Matn2 | 15 | 34.36 | 7.93 | 0.705 | 2.96E-04 |
| 1455149_at | 59009 | Sh3md2 | 8 | 63.87 | 7.94 | 0.705 | 2.99E-04 |
| 1416010_a_at | 13660 | Ehd1 | 19 | 6.30 | 8.29 | 0.705 | 2.99E-04 |
| 1439255_s_at | 83924 | Gpr137b | 13 | 13.45 | 9.04 | 0.705 | 3.00E-04 |
| 1422590_at | 12568 | Cdk5 | 5 | 23.92 | 10.43 | -0.705 | 3.03E-04 |
| 1453197_at | 75613 | Med25 | 7 | 52.13 | 10.26 | -0.704 | 3.04E-04 |
| 1441842_s_at | 69020 | 1500031N24Rik | 15 | 75.81 | 5.60 | 0.704 | 3.05E-04 |
| 1454968_at | 109065 | 1110034A24Rik | 12 | 70.29 | 6.08 | 0.704 | 3.08E-04 |
| 1425914_a_at | 78248 | Armcx1 | X | 131.26 | 10.89 | 0.704 | 3.08E-04 |
| 1416344_at | 16784 | Lamp2 | X | 35.76 | 9.81 | 0.704 | 3.11E-04 |
| 1432258_at | 77248 | 9430014N10Rik | 15 | 93.74 | 7.38 | -0.703 | 3.14E-04 |
| 1446094_at | 11906 | Atbf1 | 8 | 111.28 | 9.11 | -0.703 | 3.15E-04 |
| 1444135_at | 319832 | 6332401O19Rik | 6 | 24.90 | 8.60 | -0.703 | 3.17E-04 |
| 1435679_at | 71648 | Optn | 2 | 4.94 | 8.40 | 0.703 | 3.18E-04 |
| 1433162_at | 56193 | Plek | 11 | 16.86 | 8.58 | -0.703 | 3.19E-04 |
| 1451141_at | 228019 | BC004636 | 2 | 70.80 | 7.96 | 0.703 | 3.19E-04 |
| 1442414_at | 22644 | Rnf103 | 6 | 71.45 | 6.11 | 0.703 | 3.21E-04 |
| 1435001_at | 18786 | Plaa | 4 | 94.23 | 8.26 | 0.703 | 3.21E-04 |
| 1443433_at | 54712 | Plxnc1 | 10 | 94.30 | 5.13 | -0.703 | 3.22E-04 |
| 1439256_x_at | 83924 | Tm7sf1 | 13 | 12.71 | 8.21 | 0.702 | 3.24E-04 |
| 1428629_at | 74010 | 6330417C12Rik | 19 | 40.99 | 7.20 | 0.702 | 3.30E-04 |
| 1451503_at | 78688 | Nol3 | 8 | 107.80 | 8.19 | 0.701 | 3.33E-04 |
| 1450699_at | 20341 | Selenbp1 | 3 | 94.75 | 7.22 | 0.701 | 3.36E-04 |
| 1454103_at | 72882 | 2900022L05Rik | 11 | 69.36 | 6.27 | -0.701 | 3.36E-04 |
| 1455170_at | 66456 | 2810001G20Rik | 11 | 63.90 | 8.24 | 0.701 | 3.38E-04 |
| 1442059_at | 14359 | Fxr1h | 3 | 33.97 | 6.91 | 0.701 | 3.38E-04 |
| 1426909_at | 80914 | Uck2 | 1 | 169.15 | 9.67 | -0.701 | 3.40E-04 |
| 1454951_at | 67370 | Zfp606 | 7 | 13.08 | 6.38 | 0.701 | 3.43E-04 |
| 1422126_a_at | 67725 | Nudt13 | 14 | 21.14 | 6.43 | 0.700 | 3.51E-04 |
| 1460480_at | 71995 | 1600014E20Rik | 2 | 131.68 | 7.95 | -0.700 | 3.51E-04 |
| 1423300_at | 66980 | Zdhhc6 | 19 | 55.37 | 8.49 | 0.700 | 3.51E-04 |
| 1427936_at | 208967 | Thnsl1 | 2 | 21.14 | 7.13 | 0.700 | 3.54E-04 |
| 1433863_at | 218490 | Btf3 | 13 | 99.08 | 5.48 | 0.699 | 3.56E-04 |
| 1429936_at | 72614 | 2700059L22Rik | 9 | 50.43 | 5.26 | 0.699 | 3.57E-04 |
| 1434749_at | 216292 | BC067068 | 10 | 105.20 | 7.55 | 0.699 | 3.58E-04 |
| 1427202_at | 320204 | 4833442J19Rik | 6 | 149.10 | 6.72 | 0.699 | 3.59E-04 |
| 1418391_at | 192285 | Phf21a | 2 | 92.20 | 8.36 | 0.699 | 3.60E-04 |
| 1424462_at | 67456 | Ergic2 | 6 | 148.13 | 9.29 | 0.699 | 3.62E-04 |
| 1441205_at | 73458 | 1700055N04Rik | 19 | 3.97 | 7.87 | -0.698 | 3.69E-04 |
| 1454312_at |  | 2900097C17Rik | 2 | 156.22 | 7.91 | -0.698 | 3.72E-04 |
| 1417056_at | 19186 | Psme1 | 14 | 56.20 | 9.80 | 0.698 | 3.75E-04 |
| 1429373_x_at | 74343 | 4632407F12Rik | 3 | 90.07 | 8.21 | 0.697 | 3.79E-04 |
| 1433978_at | 68281 | 4930430F08Rik | 10 | 100.04 | 8.30 | 0.697 | 3.84E-04 |
| 1419171_at | 67698 | Tmem157 | 1 | 97.23 | 6.88 | 0.697 | 3.88E-04 |
| 1440819_s_at | 230676 | BC059842 | 4 | 118.04 | 7.59 | 0.697 | 3.88E-04 |
| 1455053_a_at | 114893 | Dcun1d1 | 3 | 35.79 | 7.28 | 0.696 | 3.91E-04 |
| 1438161_s_at | 106344 | Rfc4 | 16 | 23.11 | 5.02 | 0.696 | 3.92E-04 |
| 1456000_at | 66942 | Ddx18 | 1 | 123.45 | 6.46 | 0.696 | 3.94E-04 |
| 1427172_at | 237222 | Ofd1 | X | 162.83 | 5.25 | 0.696 | 3.94E-04 |
| 1444873_at | 215114 | Hip1 | 5 | 135.96 | 7.44 | -0.696 | 3.95E-04 |
| 1420792_at | 114673 | 4930433N12Rik | 9 | 3.13 | 4.62 | -0.696 | 3.98E-04 |
| 1424169_at | 76281 | Tax1bp3 | 11 | 72.99 | 8.73 | -0.696 | 3.99E-04 |
| 1424669_at | 68520 | Zfyve21 | 12 | 113.07 | 6.69 | 0.696 | 4.00E-04 |
| 1439699_at | 18667 | Pgr | 9 | 8.95 | 5.91 | 0.695 | 4.01E-04 |
| 1418941_at | 93893 | Pcdhb22 | 18 | 37.68 | 6.13 | 0.695 | 4.02E-04 |
| 1455611_at | 56469 | Pias1 | 9 | 62.73 | 8.21 | 0.695 | 4.03E-04 |
| 1430703_at |  | AK017437 | 11 | 99.73 | 4.50 | -0.695 | 4.04E-04 |
| 1448709_at | 93760 | Arid1a | 4 | 133.24 | 11.42 | -0.695 | 4.04E-04 |
| 1435774_at | 72057 | Phf10 | 17 | 15.08 | 8.69 | 0.695 | 4.05E-04 |
| 1417658_at | 21379 | Tbrg4 | 11 | 6.52 | 7.17 | 0.695 | 4.05E-04 |
| 1428840_s_at | 68980 | Wdr53 | 16 | 32.26 | 6.99 | 0.695 | 4.06E-04 |
| 1434004_at | 330817 | Dhps | 8 | 87.60 | 9.11 | 0.695 | 4.06E-04 |
| 1442953_at | 19775 | Xpr1 | 1 | 157.17 | 7.93 | -0.695 | 4.07E-04 |
| 1455184_at | 68473 | Mobkl1a | 5 | 89.19 | 7.65 | 0.695 | 4.10E-04 |
| 1417163_at | 63953 | Dusp10 | 1 | 185.90 | 7.31 | 0.694 | 4.13E-04 |
| 1446930_at |  | 9830127L17Rik | 16 | 92.74 | 6.66 | -0.694 | 4.14E-04 |
| 1437058_at | 230971 | Egfl3 | 4 | 153.65 | 6.96 | -0.694 | 4.18E-04 |
| 1432156_a_at | 56874 | Rnf32 | 5 | 29.55 | 9.20 | -0.694 | 4.19E-04 |
| 1416185_a_at | 11532 | Adh5 | 3 | 138.12 | 10.66 | 0.694 | 4.24E-04 |
| 1420095_s_at | 22697 | Zipro1 | 5 | 138.58 | 8.20 | 0.693 | 4.34E-04 |
| 1437926_at | 321003 | E430012M05Rik | 15 | 81.28 | 8.08 | 0.693 | 4.37E-04 |
| 1429901_at | 432450 | 6330571D19Rik | 10 | 31.41 | 8.29 | 0.692 | 4.39E-04 |
| 1424782_at | 67171 | 2610318G18Rik | 3 | 106.38 | 8.57 | 0.692 | 4.40E-04 |
| 1437334_x_at | 74108 | Parn | 16 | 13.54 | 8.73 | 0.692 | 4.42E-04 |
| 1424478_at | 67378 | Bbs2 | 8 | 96.59 | 8.82 | 0.692 | 4.47E-04 |
| 1419207_at | 22696 | Zfp37 | 4 | 61.85 | 8.50 | 0.692 | 4.47E-04 |
| 1432136_s_at | 72881 | Zdhhc4 | 5 | 144.08 | 9.52 | 0.692 | 4.47E-04 |
| 1424394_at | 114679 | Selm | 11 | 3.42 | 11.07 | 0.692 | 4.47E-04 |
| 1430811_a_at | 66977 | Cdca1 | 1 | 171.43 | 5.78 | 0.692 | 4.48E-04 |
| 1449232_at | 14460 | Gata1 | X | 7.54 | 7.48 | -0.692 | 4.48E-04 |
| 1427222_a_at | 20941 | Svp2 | 2 | 164.10 | 4.33 | -0.692 | 4.49E-04 |
| 1439661_at | 71781 | Slc16a14 | 1 | 84.90 | 8.50 | -0.691 | 4.55E-04 |
| 1440755_at | 17966 | Nbr1 | 11 | 101.44 | 6.23 | 0.691 | 4.58E-04 |
| 1423985_at | 14707 | Gng5 | 3 | 146.17 | 11.81 | -0.691 | 4.61E-04 |
| 1460588_at | 241066 | Carf | 1 | 60.21 | 7.74 | 0.691 | 4.62E-04 |
| 1417635_at | 20686 | Spa17 | 9 | 37.42 | 5.99 | 0.691 | 4.63E-04 |
| 1436206_at | 269529 | Fbxo10 | 4 | 45.05 | 10.14 | -0.691 | 4.64E-04 |
| 1438388_at | 18231 | Nxph1 | 6 | 9.20 | 4.58 | 0.690 | 4.65E-04 |
| 1453971_at | 71463 | 8430422M09Rik | 16 | 43.94 | 6.49 | -0.690 | 4.66E-04 |
| 1416158_at | 11819 | Nr2f2 | 7 | 77.50 | 7.44 | 0.690 | 4.67E-04 |
| 1428809_at | 69066 | 1810010H24Rik | 11 | 106.89 | 6.77 | 0.690 | 4.68E-04 |
| 1459256_at | 18187 | Nrp2 | 1 | 62.78 | 9.77 | -0.690 | 4.71E-04 |
| 1455860_at | 110417 | Pigh | 12 | 80.18 | 6.77 | 0.690 | 4.71E-04 |
| 1455563_at | 234374 | Ddx49 | 8 | 72.82 | 7.40 | 0.690 | 4.73E-04 |
| 1423821_at | 101118 | Tmem168 | 6 | 13.53 | 6.87 | 0.690 | 4.73E-04 |
| 1455915_at | 14426 | Galnt4 | 10 | 98.58 | 7.19 | 0.690 | 4.76E-04 |
| 1442905_at | 11843 | Arf4 | 14 | 27.48 | 8.17 | 0.689 | 4.80E-04 |
| 1421826_at | 54485 | Dll4 | 2 | 119.16 | 8.00 | -0.689 | 4.80E-04 |
| 1417030_at | 66950 | 2310028N02Rik | 1 | 193.17 | 5.47 | 0.689 | 4.84E-04 |
| 1443006_at | 27204 | Syn3 | 10 | 85.67 | 8.43 | -0.689 | 4.84E-04 |
| 1430805_s_at | 74386 | Rmi1 | 13 | 58.51 | 7.69 | 0.689 | 4.85E-04 |
| 1453566_at | 381626 | 3200001K10Rik | 5 | 28.68 | 8.58 | -0.689 | 4.85E-04 |
| 1428682_at | 232679 | Zc3hdc6 | 2 | 128.84 | 8.93 | 0.689 | 4.86E-04 |
| 1449951_at | 18038 | Nfkbil1 | 17 | 35.36 | 6.48 | 0.689 | 4.87E-04 |
| 1455392_at | 320111 | Prr18 | 17 | 8.54 | 10.27 | 0.689 | 4.92E-04 |
| 1421557_x_at | 56551 | Txn2 | 15 | 77.75 | 11.03 | -0.689 | 4.92E-04 |
| 1440291_at | 102339 | Cog4 | 2 | 146.05 | 6.12 | 0.688 | 4.95E-04 |
| 1451080_at | 230484 | Usp1 | 4 | 98.60 | 9.12 | 0.688 | 4.97E-04 |
| 1431143_x_at | 71361 | Amid | 10 | 61.20 | 7.33 | 0.688 | 4.98E-04 |
| 1445776_at |  | 4933427D06 | 7 | 47.12 | 6.82 | -0.688 | 5.00E-04 |
| 1416358_at | 68294 | 0610009O03Rik | 5 | 34.98 | 8.07 | 0.688 | 5.01E-04 |
| 1453040_at | 67062 | 2810402A17Rik | X | 133.54 | 6.31 | 0.688 | 5.03E-04 |
| 1438952_x_at | 21357 | Tarbp2 | 15 | 102.35 | 6.24 | 0.688 | 5.05E-04 |
| 1454846_at | 105372 | Utp15 | 13 | 99.02 | 8.59 | 0.687 | 5.11E-04 |
| 1432737_at |  | 2810026P18Rik | 4 | 138.42 | 7.76 | -0.687 | 5.14E-04 |
| 1428192_at | 211255 | Kbtbd7 | 14 | 79.83 | 7.65 | 0.687 | 5.15E-04 |
| 1430147_a_at | 75316 | 4930553M18Rik | 9 | 15.11 | 4.96 | 0.687 | 5.16E-04 |
| 1415992_at | 56703 | Pigo | 4 | 43.03 | 8.93 | -0.686 | 5.26E-04 |
| 1417741_at | 110095 | Pygl | 12 | 71.29 | 6.12 | 0.686 | 5.26E-04 |
| 1417246_at | 11287 | Pzp | 6 | 128.43 | 5.93 | -0.686 | 5.29E-04 |
| 1434550_at | 218734 | 3830406C13Rik | 14 | 13.14 | 6.89 | 0.686 | 5.30E-04 |
| 1433272_at | 223332 | C130037N17Rik | 15 | 8.94 | 9.11 | -0.686 | 5.33E-04 |
| 1434956_at | 77733 | Rnf170 | 8 | 27.25 | 9.19 | 0.686 | 5.34E-04 |
| 1444148_at |  | D030053O22Rik | 7 | 87.21 | 7.02 | -0.686 | 5.36E-04 |
| 1444036_at | 226849 | Ppp2r5a | 1 | 193.20 | 5.54 | 0.686 | 5.37E-04 |
| 1438729_at | 20664 | Sox1 | 8 | 12.40 | 8.89 | 0.685 | 5.38E-04 |
| 1417250_at | 19820 | Rnf12 | X | 101.15 | 9.35 | 0.685 | 5.39E-04 |
| 1425312_s_at | 52670 | D11Ertd636e | 11 | 113.56 | 7.86 | -0.685 | 5.41E-04 |
| 1439788_at | 408022 | MGC86034 | 8 | 47.66 | 5.79 | 0.685 | 5.48E-04 |
| 1454795_at | 319876 | Cobll1 | 2 | 64.93 | 7.89 | 0.685 | 5.54E-04 |
| 1448555_at | 71919 | D15Ertd682e | 15 | 97.51 | 8.79 | 0.685 | 5.54E-04 |
| 1434425_at |  | AI597080 | 3 | 93.25 | 4.69 | 0.684 | 5.55E-04 |
| 1460122_at | 233724 | Tmem41b | 7 | 117.12 | 7.93 | 0.684 | 5.58E-04 |
| 1448721_at | 52392 | C5orf30 | 1 | 99.54 | 8.87 | 0.684 | 5.63E-04 |
| 1416433_at | 19891 | Rpa2 | 4 | 132.33 | 6.80 | 0.684 | 5.69E-04 |
| 1451508_at | 71738 | Mamdc2 | 19 | 23.42 | 7.82 | 0.684 | 5.69E-04 |
| 1455195_at | 20088 | Rps24 | 14 | 25.31 | 7.36 | 0.683 | 5.73E-04 |
| 1433352_at | 77654 | C430049A07Rik | 3 | 27.31 | 7.70 | -0.683 | 5.74E-04 |
| 1433332_at | 78693 | C030004M13Rik | 17 | 9.13 | 7.29 | -0.683 | 5.74E-04 |
| 1441334_at | 22284 | Usp9x | X | 12.75 | 7.37 | 0.683 | 5.82E-04 |
| 1444007_at | 320174 | A830082K12Rik | 13 | 78.37 | 6.06 | 0.683 | 5.82E-04 |
| 1429700_at | 73176 | 3110040M04Rik | 1 | 153.04 | 6.06 | -0.683 | 5.84E-04 |
| 1424358_at | 218793 | Ube2e2 | 14 | 19.41 | 10.36 | 0.683 | 5.84E-04 |
| 1416803_at | 14231 | Fkbp7 | 2 | 76.50 | 8.06 | 0.683 | 5.86E-04 |
| 1431280_at | 103266 | AI597468 | 10 | 84.58 | 7.05 | -0.682 | 5.87E-04 |
| 1427705_a_at | 18033 | Nfkb1 | 3 | 135.25 | 9.84 | -0.682 | 5.87E-04 |
| 1443719_x_at | 72047 | Ddx42 | 11 | 106.10 | 9.47 | -0.682 | 5.90E-04 |
| 1436574_at | 70005 | 1700029I01Rik | 4 | 145.42 | 6.35 | 0.682 | 5.91E-04 |
| 1442689_at | 74211 | 1700017B05Rik | 9 | 57.11 | 7.30 | -0.682 | 5.93E-04 |
| 1451167_at | 75565 | Ccdc101 | 7 | 133.82 | 6.90 | 0.682 | 5.93E-04 |
| 1424927_at | 73690 | Glipr1 | 10 | 111.42 | 7.02 | -0.682 | 5.95E-04 |
| 1433301_at | 269639 | Zfp512 | 5 | 31.75 | 6.24 | -0.682 | 5.96E-04 |
| 1422525_at | 11958 | Atp5k | 5 | 108.86 | 13.06 | -0.682 | 5.96E-04 |
| 1455974_at | 66193 | 1110049F12Rik | 4 | 135.53 | 9.01 | -0.682 | 5.97E-04 |
| 1460370_at | 72960 | Top1mt | 15 | 75.49 | 7.93 | 0.682 | 5.98E-04 |
| 1452743_at | 59001 | Pole3 | 4 | 62.18 | 9.84 | -0.682 | 5.99E-04 |
| 1423625_a_at | 67713 | 1810055D05Rik | 3 | 33.98 | 9.03 | 0.682 | 5.99E-04 |
| 1453277_at | 70680 | 3021401N23Rik | 13 | 108.83 | 5.63 | 0.682 | 5.99E-04 |
| 1446815_at | 99349 | 1700030A21Rik | 2 | 105.82 | 7.60 | 0.682 | 6.00E-04 |
| 1429736_at | 66145 | 1110003F05Rik | 17 | 10.40 | 7.87 | 0.682 | 6.00E-04 |
| 1439706_at |  | A330106F07Rik | 2 | 23.35 | 5.43 | 0.682 | 6.02E-04 |
| 1455503_at | 212569 | 6820416H06Rik | 13 | 67.93 | 6.28 | 0.681 | 6.07E-04 |
| 1428915_at | 68346 | Sirt5 | 13 | 43.49 | 8.02 | 0.681 | 6.08E-04 |
| 1437539_at | 105787 | Prkaa1 | 15 | 5.13 | 7.38 | 0.681 | 6.11E-04 |
| 1460041_at | 396184 | Flrt1 | 19 | 7.17 | 10.50 | -0.681 | 6.11E-04 |
| 1417677_at | 13603 | Opn3 | 1 | 177.59 | 9.47 | -0.681 | 6.13E-04 |
| 1419976_s_at | 18021 | Nfatc3 | 8 | 108.65 | 6.89 | 0.681 | 6.19E-04 |
| 1454436_at | 216177 | AU041133 | 10 | 81.59 | 9.29 | -0.681 | 6.19E-04 |
| 1444446_at | 207214 | Larp4 | 15 | 99.81 | 8.68 | -0.681 | 6.19E-04 |
| 1459648_at |  | MGC37079 | 15 | 80.84 | 7.69 | -0.680 | 6.24E-04 |
| 1428889_at | 69113 | 1810020C19Rik | 2 | 93.82 | 8.65 | 0.680 | 6.29E-04 |
| 1421410_a_at | 20979 | Syt1 | 10 | 108.05 | 5.70 | 0.680 | 6.32E-04 |
| 1451741_a_at | 12572 | Cdk7 | 13 | 101.47 | 7.80 | 0.680 | 6.33E-04 |
| 1448901_at | 56264 | Cpxm1 | 2 | 130.22 | 6.92 | 0.680 | 6.34E-04 |
| 1451747_a_at | 67526 | Apg12 | 18 | 46.89 | 9.99 | -0.680 | 6.38E-04 |
| 1436641_at |  | AI415730 | 19 | 4.78 | 6.39 | 0.679 | 6.39E-04 |
| 1424388_at | 76779 | Cluap1 | 16 | 3.93 | 8.74 | 0.679 | 6.39E-04 |
| 1455097_at | 73770 | 4833422F06Rik | 15 | 7.83 | 7.67 | 0.679 | 6.43E-04 |
| 1457373_at | 227485 | Cdh19 | 1 | 112.78 | 7.52 | 0.679 | 6.50E-04 |
| 1449583_at | 93891 | Pcdhb20 | 18 | 37.67 | 8.64 | 0.679 | 6.51E-04 |
| 1420054_s_at | 228875 | Slc35c2 | 2 | 165.10 | 7.50 | 0.679 | 6.53E-04 |
| 1418034_at | 69527 | Mrps9 | 1 | 42.96 | 8.53 | 0.679 | 6.53E-04 |
| 1450469_at | 13195 | Ddc8 | 11 | 118.19 | 8.34 | -0.679 | 6.55E-04 |
| 1417894_at | 54672 | Gpr97 | 8 | 97.57 | 7.62 | -0.679 | 6.56E-04 |
| 1455769_at | 66299 | 2610019N06Rik | 9 | 7.18 | 5.51 | 0.678 | 6.58E-04 |
| 1447553_x_at | 320360 | E130307J04Rik | 7 | 116.19 | 5.27 | 0.678 | 6.60E-04 |
| 1458474_at | 216169 | D10Bwg1364e | 10 | 80.05 | 9.66 | -0.678 | 6.62E-04 |
| 1436107_at | 76522 | Lsm8 | 6 | 18.81 | 6.92 | 0.678 | 6.63E-04 |
| 1449050_at | 19687 | Recc1 | 5 | 65.65 | 7.58 | 0.678 | 6.68E-04 |
| 1447595_x_at | 69108 | 1810012K16Rik | 8 | 23.51 | 5.85 | -0.678 | 6.71E-04 |
| 1447914_x_at | 72446 | 2600010E01Rik | 2 | 101.55 | 5.15 | 0.678 | 6.72E-04 |
| 1436661_at | 269109 | Dpp10 | 1 | 125.23 | 10.32 | 0.678 | 6.72E-04 |
| 1447606_x_at | 66333 | Aqp11 | 7 | 104.87 | 9.67 | -0.677 | 6.81E-04 |
| 1417233_at | 72170 | Chchd4 | 6 | 91.41 | 11.13 | -0.677 | 6.85E-04 |
| 1458656_at | 101821 | AW050198 | 7 | 54.26 | 7.67 | 0.677 | 6.93E-04 |
| 1429801_at | 71901 | 2310028H24Rik | 4 | 41.47 | 6.69 | 0.677 | 6.93E-04 |
| 1449880_s_at | 12096 | Bglap1 | 3 | 88.18 | 9.20 | -0.677 | 6.94E-04 |
| 1435914_at | 20185 | Ncor1 | 11 | 62.22 | 8.43 | 0.676 | 6.95E-04 |
| 1460299_at | 15285 | Hlxb9 | 5 | 29.80 | 5.34 | -0.676 | 6.95E-04 |
| 1425473_at | 234959 | Crsp6 | 9 | 15.07 | 9.70 | 0.676 | 6.97E-04 |
| 1428839_at | 68980 | Wdr53 | 16 | 32.26 | 8.09 | 0.676 | 6.98E-04 |
| 1460587_at | 320478 | B230215L15Rik | 3 | 34.58 | 8.29 | 0.676 | 7.05E-04 |
| 1439308_at | 76615 | Got1l1 | 8 | 28.31 | 11.03 | -0.676 | 7.06E-04 |
| 1426210_x_at | 235587 | Parp3 | 9 | 106.37 | 4.97 | 0.676 | 7.06E-04 |
| 1439638_at | 59079 | Erbb2ip | 13 | 104.62 | 7.23 | 0.676 | 7.10E-04 |
| 1444086_at | 210356 | Nap5 | 1 | 127.81 | 6.25 | 0.676 | 7.10E-04 |
| 1439481_at | 226432 | Ipo9 | 1 | 137.28 | 9.32 | 0.676 | 7.10E-04 |
| 1448687_at | 67389 | C1qdc2 | 4 | 155.34 | 9.46 | -0.675 | 7.14E-04 |
| 1424637_s_at | 67163 | Ccdc47 | 11 | 106.06 | 11.11 | 0.675 | 7.15E-04 |
| 1448398_s_at | 19934 | Rpl22 | 4 | 151.71 | 10.37 | 0.675 | 7.16E-04 |
| 1437055_x_at | 66868 | Mfsd1 | 3 | 67.41 | 7.50 | 0.675 | 7.17E-04 |
| 1455475_at | 269423 | 3110057O12Rik | 3 | 40.74 | 7.98 | 0.675 | 7.24E-04 |
| 1458564_at | 72670 | 2810029C07Rik | 19 | 42.96 | 5.67 | -0.675 | 7.28E-04 |
| 1430816_at | 68053 | 3110003A22Rik | 4 | 6.12 | 9.24 | -0.675 | 7.31E-04 |
| 1423172_at | 17957 | Napb | 2 | 148.52 | 12.10 | 0.674 | 7.34E-04 |
| 1441909_s_at | 214112 | 9530066K23Rik | 11 | 45.96 | 5.32 | 0.674 | 7.35E-04 |
| 1436247_at | 70422 | Ints2 | 11 | 86.02 | 7.27 | 0.674 | 7.36E-04 |
| 1434191_at | 319660 | Tmem195 | 12 | 38.31 | 7.59 | 0.674 | 7.42E-04 |
| 1416338_at | 20405 | Sh3gl1 | 17 | 56.16 | 9.71 | -0.674 | 7.45E-04 |
| 1448382_at | 74147 | Ehhadh | 16 | 21.76 | 5.24 | 0.674 | 7.46E-04 |
| 1448403_at | 107045 | Lars | 18 | 42.36 | 9.33 | 0.674 | 7.47E-04 |
| 1455451_at | 233529 | Kctd14 | 7 | 104.61 | 4.46 | 0.673 | 7.55E-04 |
| 1433756_at | 74648 | S100pbp | 4 | 128.83 | 10.06 | 0.673 | 7.58E-04 |
| 1417637_a_at | 15353 | Hmg20b | 10 | 80.81 | 7.59 | 0.673 | 7.59E-04 |
| 1435316_at | 26443 | Psma6 | 12 | 56.52 | 4.56 | 0.673 | 7.60E-04 |
| 1451001_at | 67507 | 1700019N19Rik | 19 | 58.86 | 6.28 | -0.673 | 7.61E-04 |
| 1455607_at | 72780 | Rspo3 | 10 | 29.17 | 7.41 | 0.673 | 7.61E-04 |
| 1453945_at | 70090 | 2310005E17Rik | 13 | 99.19 | 4.81 | -0.673 | 7.69E-04 |
| 1441284_at | 23988 | Pin1 | 9 | 20.45 | 6.84 | 0.673 | 7.74E-04 |
| 1427855_at | 243453 | Igk-V8 | 6 | 68.94 | 7.91 | -0.673 | 7.74E-04 |
| 1454576_at | 218639 | Arfrp2 | 13 | 114.94 | 7.25 | -0.673 | 7.74E-04 |
| 1427124_at | 67333 | 1700054C12Rik | 2 | 129.63 | 7.04 | 0.672 | 7.78E-04 |
| 1441936_x_at | | AB041803 | 2 | 19.27 | 5.74 | 0.672 | 7.79E-04 |
| 1416874_a_at | 54624 | Paf1 | 7 | 29.18 | 8.18 | 0.672 | 7.79E-04 |
| 1432993_at | 75774 | 4930403L11Rik | 10 | 66.95 | 6.79 | -0.672 | 7.86E-04 |
| 1444174_at | 30806 | Adamts8 | 9 | 30.75 | 6.37 | 0.672 | 7.87E-04 |
| 1418512_at | 56274 | Stk3 | 15 | 34.81 | 8.21 | 0.672 | 7.87E-04 |
| 1442808_at |  | 1810005K13Rik | 18 | 80.42 | 6.83 | -0.672 | 7.89E-04 |
| 1445054_at | 97268 | C230066G23Rik | 17 | 88.87 | 8.32 | -0.672 | 7.92E-04 |
| 1455635_at | 320929 | 4732460I02Rik | X | 54.63 | 5.43 | 0.672 | 7.94E-04 |
| 1425058_at | 224691 | Zfp472 | 17 | 33.12 | 7.31 | 0.672 | 7.95E-04 |
| 1436104_a_at | 72315 | 2310015A05Rik | 16 | 17.65 | 7.93 | 0.671 | 7.99E-04 |
| 1428390_at | 72515 | Wdr43 | 17 | 72.00 | 9.69 | -0.671 | 8.03E-04 |
| 1416859_at | 30795 | Fkbp3 | 12 | 66.16 | 10.81 | 0.671 | 8.05E-04 |
| 1432699_at | 72845 | 2900024I21Rik | 14 | 62.07 | 7.20 | -0.671 | 8.11E-04 |
| 1452076_at | 66701 | 4633402N23Rik | 10 | 127.65 | 9.05 | -0.671 | 8.15E-04 |
| 1416216_at | 19707 | Reps1 | 10 | 17.84 | 8.97 | 0.671 | 8.17E-04 |
| 1421261_at | 16891 | Lipg | 18 | 75.10 | 7.94 | -0.670 | 8.24E-04 |
| 1457217_at | 18483 | Palm | 10 | 79.26 | 8.48 | -0.670 | 8.28E-04 |
| 1429778_at | 71648 | Optn | 2 | 4.94 | 7.76 | 0.670 | 8.28E-04 |
| 1432967_at | 233871 | Atxn2l | 7 | 133.65 | 5.50 | -0.670 | 8.31E-04 |
| 1431295_a_at | 71116 | Stx18 | 5 | 38.52 | 6.78 | 0.670 | 8.33E-04 |
| 1446822_at | 11481 | Acvrinp1 | 5 | 20.10 | 7.82 | -0.670 | 8.33E-04 |
| 1427390_at | 232946 | Bloc1s3 | 7 | 20.09 | 9.66 | -0.670 | 8.34E-04 |
| 1451535_at | 218624 | Il31ra | 13 | 113.31 | 7.36 | -0.670 | 8.35E-04 |
| 1455488_at | 230376 | 6230416J20Rik | 4 | 86.23 | 6.15 | 0.670 | 8.36E-04 |
| 1416282_at | 19182 | Psmc3 | 2 | 90.90 | 11.09 | 0.670 | 8.40E-04 |
| 1460087_at | 217370 | BC017643 | 11 | 121.09 | 6.73 | -0.669 | 8.46E-04 |
| 1431510_s_at | 67899 | 2010110K16Rik | 9 | 117.97 | 8.26 | 0.669 | 8.47E-04 |
| 1436469_at | 26992 | Brd7 | 8 | 90.85 | 6.50 | 0.669 | 8.47E-04 |
| 1424432_at | 226122 | Ubtd1 | 19 | 42.11 | 10.02 | -0.669 | 8.47E-04 |
| 1450704_at | 16147 | Ihh | 1 | 74.99 | 7.92 | -0.669 | 8.48E-04 |
| 1435042_at |  | 9130004C02Rik | 9 | 25.06 | 6.94 | 0.669 | 8.52E-04 |
| 1452224_at | 338467 | Zcwcc3 | 16 | 93.88 | 7.90 | 0.669 | 8.54E-04 |
| 1433034_at | 433501 | LOC433501 | 2 | 164.35 | 7.31 | -0.669 | 8.55E-04 |
| 1446049_at | 58250 | Chst11 | 10 | 82.66 | 7.39 | -0.669 | 8.58E-04 |
| 1422193_at | 14919 | Gucy2e | 11 | 69.04 | 7.00 | -0.669 | 8.60E-04 |
| 1452217_at | 66395 | Ahnak | 19 | 9.09 | 9.29 | 0.669 | 8.61E-04 |
| 1448055_at | 319862 | E230011A21Rik | 3 | 108.68 | 6.83 | -0.668 | 8.70E-04 |
| 1437194_x_at | 71735 | 1200011O22Rik | 5 | 136.60 | 8.29 | 0.668 | 8.72E-04 |
| 1427258_at | 21848 | Trim24 | 6 | 37.92 | 9.02 | 0.668 | 8.73E-04 |
| 1431435_at | 76142 | Ppp1r14c | 10 | 6.98 | 6.04 | -0.668 | 8.75E-04 |
| 1427934_at | 108755 | 2610208E05Rik | 4 | 32.89 | 7.78 | 0.668 | 8.76E-04 |
| 1420486_at | 70078 | Nol7 | 13 | 43.50 | 10.30 | 0.668 | 8.81E-04 |
| 1425463_at | 14465 | Gata6 | 18 | 11.06 | 6.07 | -0.667 | 8.90E-04 |
| 1451266_at | 28028 | Mrpl50 | 4 | 49.53 | 8.02 | 0.667 | 8.93E-04 |
| 1432221_at | 76679 | 5330417H12Rik | 7 | 114.77 | 8.58 | -0.667 | 8.95E-04 |
| 1423548_s_at | 66366 | Ergic3 | 2 | 155.84 | 9.57 | 0.667 | 8.99E-04 |
| 1437108_at | 78651 | Lsm6 | 8 | 81.33 | 7.94 | 0.667 | 9.01E-04 |
| 1418571_at | 27279 | Tnfrsf12a | 17 | 23.81 | 9.15 | -0.667 | 9.05E-04 |
| 1424162_at | 72169 | Trim29 | 9 | 43.14 | 8.27 | -0.667 | 9.09E-04 |
| 1426594_at | 232288 | Frmd4b | 6 | 97.24 | 7.27 | 0.666 | 9.17E-04 |
| 1428557_a_at | 72085 | Osgepl1 | 1 | 53.38 | 8.45 | 0.666 | 9.19E-04 |
| 1417594_at | 56278 | Gkap1 | 13 | 58.33 | 9.51 | 0.666 | 9.20E-04 |
| 1442397_at | 74164 | Nfx1 | 4 | 40.96 | 7.64 | 0.666 | 9.22E-04 |
| 1435972_at | 12380 | Cast | 13 | 74.83 | 7.44 | 0.666 | 9.26E-04 |
| 1440272_at | 13665 | Eif2s1 | 12 | 79.99 | 8.13 | 0.666 | 9.28E-04 |
| 1420754_at | 22130 | Ttf1 | 2 | 28.94 | 9.63 | -0.666 | 9.30E-04 |
| 1444644_x_at | 69694 | Tatdn1 | 15 | 58.74 | 8.44 | 0.666 | 9.30E-04 |
| 1425912_at | 214552 | BC027092 | 9 | 45.60 | 8.61 | -0.666 | 9.31E-04 |
| 1431961_at | 76088 | Dock8 | 19 | 25.17 | 7.85 | -0.666 | 9.33E-04 |
| 1428678_s_at | 71968 | 2410008B13Rik | 7 | 88.04 | 7.53 | 0.665 | 9.39E-04 |
| 1460583_at | 66964 | Golt1b | 6 | 142.35 | 6.41 | 0.665 | 9.42E-04 |
| 1432518_at | 78109 | 4930430J20Rik | 4 | 107.50 | 7.49 | -0.665 | 9.46E-04 |
| 1434677_at | 246694 | Hps5 | 7 | 54.02 | 5.61 | 0.664 | 9.64E-04 |
| 1426625_at | 78834 | Zfp623 | 15 | 75.78 | 9.60 | -0.664 | 9.65E-04 |
| 1448933_at | 93888 | Pcdhb17 | 18 | 37.65 | 8.89 | 0.664 | 9.68E-04 |
| 1430023_at | 71242 | 5133400G04Rik | 18 | 35.82 | 6.61 | 0.664 | 9.73E-04 |
| 1452738_at | 69106 | Stoml1 | 9 | 58.11 | 9.23 | 0.664 | 9.74E-04 |
| 1452884_at | 72193 | Sfrs2ip | 15 | 96.25 | 8.34 | 0.664 | 9.76E-04 |
| 1442154_at | 22184 | U2af1-rs2 | X | 160.39 | 7.78 | 0.664 | 9.80E-04 |
| 1417983_a_at | 70620 | Ube2v2 | 16 | 19.03 | 9.66 | 0.664 | 9.81E-04 |
| 1435776_at | 69549 | C1orf53 | 1 | 140.75 | 7.00 | 0.664 | 9.82E-04 |
| 1435273_at | 70560 | Wars2 | 3 | 99.02 | 7.78 | -0.663 | 9.89E-04 |
| 1439504_s_at | 22690 | Zfp28 | 7 | 6.35 | 6.20 | 0.663 | 9.93E-04 |
| 1446141_at | 21808 | Tgfb2 | 1 | 188.50 | 8.25 | -0.663 | 9.94E-04 |
| 1441837_at | 11444 | Chrnb2 | 3 | 89.56 | 7.32 | 0.663 | 9.96E-04 |
| 1429690_at | 74149 | 1300003B13Rik | 17 | 22.59 | 9.12 | 0.663 | 9.99E-04 |
