# Supplementary material for: Genetic Analysis of the Neurosteroid Deoxycorticosterone and Its Relation to Alcohol Phenotypes: Identification of QTLs and Downstream Gene Regulation
Source: PLoS One. 2011 Apr 8;6(4):e18405. doi: 10.1371/journal.pone.0018405 (PMC3072994; doi:10.1371/journal.pone.0018405)
Supplement: Table S7 — Pearson's correlations of the log-transformed DOC data are reported. HIC: handling-induced convulsions. (DOC) [file pone.0018405.s009.doc]

**Table S7.** Genetic correlations between dexamethasone suppression of cerebral cortical or plasma DOC levels and behavioral phenotypes across the BXD strains.

|  |  |  | **Cerebral cortex** | | | **Plasma** | | |
| --- | --- | --- | --- | --- | --- | --- | --- | --- |
| **Phenotypes** | **Reference** | **GN ID** | **r** | **p** | **n** | **r** | **p** | **n** |
|  |  |  |  |  |  |  |  |  |
| Ethanol-induced ataxia, 2 g/kg, ip | (Phillips et al. 1996) | 10498 | -0.58 | 0.011 | 18 |  |  |  |
| Ethanol-induced ataxia, 2 g/kg, ip (day 3 – day 2 in ethanol group) | (Phillips et al. 1996) | 10497 | -0.54 | 0.019 | 18 |  |  |  |
| Ethanol-induced hypothermia, 2 g/kg | (Crabbe et al. 1996) | 10087 | -0.66 | 0.002 | 18 | -0.73 | 0.0002 | 19 |
| Ethanol-induced hypothermia: average response at 30 and 60 min, 2 g/kg | (Crabbe et al. 1994) | 10067 | 0.66 | 0.004 | 16 | 0.74 | 0.0004 | 17 |
| Ethanol-induced hypothermia, 3 g/kg | (Crabbe et al. 1996) | 10086 | -0.57 | 0.013 | 18 | -0.53 | 0.018 | 19 |
| Ethanol-induced hypothermia: average response at 30 and 60 min, 3 g/kg | (Crabbe et al. 1994) | 10068 | 0.58 | 0.017 | 16 | 0.58 | 0.014 | 17 |
| Ethanol-induced locomotion: 0-5 min | (Demarest et al. 2001) | 10790 |  |  |  | -0.51 | 0.021 | 20 |
| Ethanol-induced locomotion: 5-10 min | (Demarest et al. 2001) | 10791 | -0.60 | 0.006 | 19 | -0.60 | 0.004 | 20 |
| Ethanol-induced locomotion: 10-15 min | (Demarest et al. 2001) | 10792 | -0.60 | 0.005 | 19 | -0.55 | 0.011 | 20 |
| Ethanol-induced locomotion: 15-20 min | (Demarest et al. 2001) | 10793 | -0.48 | 0.037 | 19 | -0.46 | 0.041 | 20 |
| Ethanol-induced locomotion, 2 g/kg (day 11 - day 2 in saline group) | (Phillips et al. 1996) | 10495 | -0.56 | 0.014 | 18 |  |  |  |
| Ethanol-induced locomotion, 2 g/kg (day 3 - day 2, in ethanol group) | (Phillips et al. 1996) | 10494 | -0.55 | 0.016 | 18 | -0.54 | 0.015 | 19 |
| Ethanol-induced locomotion, 2 g/kg (difference in acute locomotion 1-5 min after injection in chronic ethanol sensitized group) | (Phillips et al. 1995) | 10485 | -0.56 | 0.024 | 16 | -0.64 | 0.005 | 17 |
| Ethanol acceptance relative to water in two-bottle choice test | (Crabbe et al. 1983) | 10073 |  |  |  | 0.57 | 0.039 | 13 |
| Ethanol open field activity, relative to saline | (Crabbe et al. 1983) | 10077 |  |  |  | -0.49 | 0.045 | 17 |
| Restraint stress + ethanol 1.8 g/kg, ip, time in open quadrants of elevated zero maze, 10 min, males | Cook et al, unpublished | 12438 | -0.37 | 0.025 | 37 |  |  |  |
| Restraint stress + ethanol 1.8 g/kg, ip, time in open quadrants of elevated zero maze, 1^st^ 5 min, males | Cook et al, unpublished | 12436 | -0.35 | 0.031 | 37 |  |  |  |
|  |  |  |  |  |  |  |  |  |
| Anxiety: time in open quadrants of elevated zero maze, first 5 min, males | Cook et al, unpublished | 12346 | 0.44 | 0.004 | 40 | 0.43 | 0.003 | 45 |
| Anxiety: time in open quadrants of elevated zero maze, 10 min, males | Cook et al, unpublished | 12348 | 0.38 | 0.015 | 40 | 0.38 | 0.010 | 45 |
| Anxiety: time in open quadrants of elevated zero maze, last 5 min, males | Cook et al, unpublished | 12347 |  |  |  | 0.30 | 0.043 | 45 |
| Anxiety: activity in closed quadrants of elevated zero maze, first 5 min, males | Cook et al, unpublished | 12352 |  |  |  | -0.30 | 0.044 | 45 |
| Anxiety: % time in light compartment, light-dark box test | (Yang et al. 2008) | 10907 | -0.68 | 0.027 | 10 |  |  |  |
| Anxiety: locomotion in light compartment, light-dark box test | (Yang et al. 2008) | 10904 | -0.67 | 0.031 | 10 |  |  |  |
| Anxiety: transitions between light and dark sides, light-dark box test | (Philip et al. 2010) | 11391 | -0.35 | 0.039 | 36 |  |  |  |
| Anxiety: time in the middle of elevated plus maze, males | (Philip et al. 2010) | 11467 |  |  |  | 0.38 | 0.013 | 41 |
| Anxiety: % entries into closed arms of elevated plus maze, males | (Philip et al. 2010) | 11456 |  |  |  | -0.32 | 0.038 | 41 |
| Open field behavior, locomotion in the center, 0-60 min, beam breaks, males | (Philip et al. 2010) | 11500 |  |  |  | 0.31 | 0.043 | 42 |
| Open field behavior, locomotion in the center, 15-30 min, beam breaks, males | (Philip et al. 2010) | 11507 |  |  |  | 0.31 | 0.045 | 42 |
| Open field behavior, locomotion in the center, 30-45 min, beam breaks, males | (Philip et al. 2010) | 11508 |  |  |  | 0.37 | 0.014 | 43 |
| Open field behavior, locomotion in the center, 0-60 min, cm, males | (Philip et al. 2010) | 11501 |  |  |  | 0.32 | 0.039 | 42 |
| Open field behavior, locomotion in the center, 0-15 min, cm, males | (Philip et al. 2010) | 11510 |  |  |  | 0.32 | 0.040 | 42 |
| Open field behavior, locomotion in the center, 15-30 min, cm, males | (Philip et al. 2010) | 11511 |  |  |  | 0.33 | 0.031 | 42 |
| Open field behavior, locomotion in the center, 30-45 min, cm, males | (Philip et al. 2010) | 11512 |  |  |  | 0.37 | 0.014 | 42 |
| Open field behavior, duration in the center | (Brigman et al. 2009) | 11015 |  |  |  | 0.51 | 0.035 | 17 |
| Fear conditioning response, activity after 1^st^ tone shock pairing, males | (Philip et al. 2010) | 11400 | -0.42 | 0.011 | 36 |  |  |  |
| Fear conditioning response, activity after 2^nd^ tone shock pairing, males | (Philip et al. 2010) | 11401 | -0.39 | 0.016 | 36 |  |  |  |
| Fear conditioning response, activity after 3^rd^ tone shock pairing, males | (Philip et al. 2010) | 11402 | -0.34 | 0.043 | 36 |  |  |  |
| Fear conditioning response, activity suppression after 3^rd^ tone shock pairing, males | (Philip et al. 2010) | 11403 |  |  |  | -0.34 | 0.029 | 41 |
| Fear conditioning response, activity in altered context during presentation of cue, males | (Philip et al. 2010) | 11395 | -0.38 | 0.020 | 36 |  |  |  |
| Acoustic startle response, baseline, males | (Philip et al. 2010) | 11421 | 0.39 | 0.019 | 36 | 0.54 | 0.0002 | 40 |
| Acoustic startle response, prepulse inhibition at 70 db, males | (Philip et al. 2010) | 11426 | 0.36 | 0.030 | 36 |  |  |  |
| Acoustic startle response, % response at 70 db, males | (Philip et al. 2010) | 11429 | -0.36 | 0.030 | 36 |  |  |  |
| Acoustic startle response, prepulse inhibition to 110 db | (McCaughran et al. 1999) | 10399 |  |  |  | 0.55 | 0.026 | 16 |
|  |  |  |  |  |  |  |  |  |
| Depression: time immobile in Porsolt test, males | (Philip et al. 2010) | 11306 | -0.44 | 0.013 | 30 |  |  |  |
|  |  |  |  |  |  |  |  |  |
| Cocaine (15 mg/kg, ip) exploratory activity | (Jones et al. 1999) | 10301 | -0.63 | 0.007 | 16 | -0.69 | 0.002 | 17 |
| Cocaine (30 mg/kg, ip) exploratory activity | (Jones et al. 1999) | 10310 |  |  |  | -0.53 | 0.029 | 17 |
|  |  |  |  |  |  |  |  |  |
| Chlordiazepoxide locomotor response, 10-15 min after 10 mg/kg | (Demarest et al. 1999) | 10122 | -0.54 | 0.020 | 18 |  |  |  |
| Chlordiazepoxide locomotor response, 5-20 min after 10 mg/kg | (Demarest et al. 1999) | 10124 | -0.50 | 0.032 | 18 |  |  |  |
| Chlordiazepoxide locomotor response, 15-20 min after 10 mg/kg | (Demarest et al. 1999) | 10123 | -0.50 | 0.033 | 18 |  |  |  |
|  |  |  |  |  |  |  |  |  |
| Methamphetamine 16 mg/kg, ip, climbing scores | (Grisel et al. 1997) | 10173 | 0.49 | 0.036 | 18 |  |  |  |
|  |  |  |  |  |  |  |  |  |
| Nitrous oxide withdrawal HIC (area under the curve) | (Belknap et al. 1993) | 10025 | 0.60 | 0.013 | 16 | 0.54 | 0.024 | 17 |
| Nitrous oxide withdrawal HIC (peak level) | (Belknap et al. 1993) | 10026 | 0.59 | 0.015 | 16 | 0.55 | 0.021 | 17 |
| Nitrous oxide withdrawal HIC (difference between treated and baseline untreated | (Belknap et al. 1993) | 10027 | 0.54 | 0.028 | 16 | 0.49 | 0.047 | 17 |
|  |  |  |  |  |  |  |  |  |
| Learning and memory: latency to reach platform in Morris water maze, test 1 | (Milhaud et al. 2002) | 10413 | -0.67 | 0.002 | 17 | -0.66 | 0.002 | 18 |
| Learning and memory: latency to reach platform in Morris water maze, test 2 | (Milhaud et al. 2002) | 10414 | -0.56 | 0.018 | 17 | -0.54 | 0.018 | 18 |
| Learning and memory: latency to reach platform in Morris water maze, test 3 | (Milhaud et al. 2002) | 10415 | -0.54 | 0.025 | 17 | -0.49 | 0.036 | 18 |
|  |  |  |  |  |  |  |  |  |
| Hypothalamus volume | (Badea et al. 2009) | 10925 | 0.90 | 0.001 | 8 | 0.92 | 7 e-6 | 11 |
| Cerebral cortex volume | (Gaglani et al. 2009) | 10997 | 0.36 | 0.032 | 35 | 0.35 | 0.029 | 39 |
| Cerebral cortex volume, bilateral | (Gaglani et al. 2009) | 10995 |  |  |  | 0.36 | 0.025 | 39 |
| Dorsal thalamus volume | (Dong et al. 2007) | 10738 | 0.47 | 0.036 | 20 | 0.60 | 0.002 | 23 |
| Laterodorsal nucleus of thalamus volume | (Badea et al. 2009) | 10935 |  |  |  | 0.71 | 0.012 | 11 |
| Ventral thalamic nuclei volume | (Badea et al. 2009) | 10945 |  |  |  | 0.69 | 0.017 | 11 |
| Hippocampus volume | (Badea et al. 2009) | 10895 |  |  |  | 0.67 | 0.021 | 11 |
| Dorsal lateral geniculate nucleus volume | (Seecharan et al. 2003) | 10595 |  |  |  | 0.46 | 0.031 | 22 |
| Cerebellum weight | (Airey et al. 2001) | 10001 |  |  |  | 0.50 | 0.021 | 21 |
| Cerebellum internal granule layer | (Airey et al. 2001) | 10006 |  |  |  | 0.51 | 0.029 | 18 |
| Olfactory bulb weight | (Williams et al. 2001) | 10645 |  |  |  | 0.46 | 0.027 | 23 |
| Brain weight | (Williams et al. 2001) | 10646 |  |  |  | 0.52 | 0.010 | 23 |
| Brain weight | (Airey et al. 2001) | 10003 |  |  |  | 0.53 | 0.011 | 21 |
| Brain weight | (Lu et al. 2001) | 10379 |  |  |  | 0.50 | 0.015 | 23 |
| Brain weight | (Seecharan et al. 2003) | 10594 |  |  |  | 0.51 | 0.029 | 18 |
| Brain weight | (Peirce et al. 2003) | 10462 |  |  |  | 0.45 | 0.038 | 21 |
| cAMP accumulation in cerebellum | (Kirstein et al. 2002) | 10352 |  |  |  | -0.48 | 0.028 | 21 |
| Pain response, chemical nociception, acetic acid writhing test | (Quock et al. 1996) | 10918 | 0.55 | 0.034 | 15 | 0.51 | 0.043 | 16 |
| Preference for cinnamon-flavored food | (Bolivar and Flaherty 2004) | 10782 | -0.50 | 0.046 | 16 | -0.57 | 0.013 | 18 |
| Time on accelerating rotarod, mean over 3 trials | (Kempermann and Gage 2002) | 10824 |  |  |  | 0.79 | 0.009 | 9 |
| Raffinose bitter taste response (% consumption) | (Lush 1986) | 10382 |  |  |  | -0.68 | 0.012 | 12 |
| Glycine sweet taste response (% consumption) | (Lush and Holland 1988) | 10386 |  |  |  | -0.59 | 0.024 | 14 |
| Saccharin consumption (two-bottle choice) | (Phillips et al. 1991) | 10471 |  |  |  | 0.52 | 0.031 | 17 |
| Dopamine transporter SLC6A3 protein density in nucleus accumbens, males | (Jones et al. 1999) | 10273 |  |  |  | -0.56 | 0.047 | 13 |
| Protein kinase C activity for cytosolic cortex | (Wehner et al. 1990) | 10626 |  |  |  | -0.71 | 0.047 | 8 |
| Visual cortex activity | (Heimel et al. 2008) | 11286 |  |  |  | 0.70 | 0.014 | 11 |
| Neurogenesis, BrdU-labeled cells in the rostral migratory stream, males | (Philip et al. 2010) | 11556 | 0.47 | 0.013 | 26 |  |  |  |

**References**

Airey DC, Lu L, Williams RW (2001) Genetic control of the mouse cerebellum: identification of quantitative trait loci modulating size and architecture. J Neurosci 21: 5099-109

Badea A, Johnson GA, Williams RW (2009) Genetic dissection of the mouse brain using high-field magnetic resonance microscopy. Neuroimage 45: 1067-79

Belknap JK, Metten P, Helms ML, O'Toole LA, Angeli-Gade S, Crabbe JC, Phillips TJ (1993) Quantitative trait loci (QTL) applications to substances of abuse: physical dependence studies with nitrous oxide and ethanol in BXD mice. Behav Genet 23: 213-22

Bolivar VJ, Flaherty L (2004) Genetic control of novel food preference in mice. Mamm Genome 15: 193-8

Brigman JL, Mathur P, Lu L, Williams RW, Holmes A (2009) Genetic relationship between anxiety-related and fear-related behaviors in BXD recombinant inbred mice. Behav Pharmacol 20: 204-9

Crabbe JC, Belknap JK, Mitchell SR, Crawshaw LI (1994) Quantitative trait loci mapping of genes that influence the sensitivity and tolerance to ethanol-induced hypothermia in BXD recombinant inbred mice. J Pharmacol Exp Ther 269: 184-192

Crabbe JC, Kosobud A, Young ER, Janowsky JS (1983) Polygenic and single-gene determination of responses to ethanol in BXD/Ty recombinant inbred mouse strains. Neurobehav Toxicol Teratol 5: 181-7

Crabbe JC, Phillips TJ, Gallaher EJ, Crawshaw LI, Mitchell SR (1996) Common genetic determinants of the ataxic and hypothermic effects of ethanol in BXD/Ty recombinant inbred mice: genetic correlations and quantitative trait loci. J Pharmacol Exp Ther 277: 624-632

Demarest K, Koyner J, McCaughran J, Jr., Cipp L, Hitzemann R (2001) Further characterization and high-resolution mapping of quantitative trait loci for ethanol-induced locomotor activity. Behav Genet 31: 79-91

Demarest K, McCaughran J, Jr., Mahjubi E, Cipp L, Hitzemann R (1999) Identification of an acute ethanol response quantitative trait locus on mouse chromosome 2. J Neurosci 19: 549-61

Dong H, Martin MV, Colvin J, Ali Z, Wang L, Lu L, Williams RW, Rosen GD, Csernansky JG, Cheverud JM (2007) Quantitative trait loci linked to thalamus and cortex gray matter volumes in BXD recombinant inbred mice. Heredity 99: 62-9

Gaglani SM, Lu L, Williams RW, Rosen GD (2009) The genetic control of neocortex volume and covariation with neocortical gene expression in mice. BMC Neurosci 10: 44

Grisel JE, Belknap JK, O'Toole LA, Helms ML, Wenger CD, Crabbe JC (1997) Quantitative trait loci affecting methamphetamine responses in BXD recombinant inbred mouse strains. J Neurosci 17: 745-54

Heimel JA, Hermans JM, Sommeijer JP, Levelt CN (2008) Genetic control of experience-dependent plasticity in the visual cortex. Genes Brain Behav 7: 915-23

Jones BC, Tarantino LM, Rodriguez LA, Reed CL, McClearn GE, Plomin R, Erwin VG (1999) Quantitative-trait loci analysis of cocaine-related behaviours and neurochemistry. Pharmacogenetics 9: 607-17

Kempermann G, Gage FH (2002) Genetic determinants of adult hippocampal neurogenesis correlate with acquisition, but not probe trial performance, in the water maze task. Eur J Neurosci 16: 129-36

Kirstein SL, Davidson KL, Ehringer MA, Sikela JM, Erwin VG, Tabakoff B (2002) Quantitative trait loci affecting initial sensitivity and acute functional tolerance to ethanol-induced ataxia and brain cAMP signaling in BXD recombinant inbred mice. J Pharmacol Exp Ther 302: 1238-45

Lu L, Airey DC, Williams RW (2001) Complex trait analysis of the hippocampus: mapping and biometric analysis of two novel gene loci with specific effects on hippocampal structure in mice. J Neurosci 21: 3503-14

Lush IE (1986) The genetics of tasting in mice. IV. The acetates of raffinose, galactose and beta-lactose. Genet Res 47: 117-23

Lush IE, Holland G (1988) The genetics of tasting in mice. V. Glycine and cycloheximide. Genet Res 52: 207-12

McCaughran J, Jr., Bell J, Hitzemann R (1999) On the relationships of high-frequency hearing loss and cochlear pathology to the acoustic startle response (ASR) and prepulse inhibition of the ASR in the BXD recombinant inbred series. Behav Genet 29: 21-30

Milhaud JM, Halley H, Lassalle JM (2002) Two QTLs located on chromosomes 1 and 5 modulate different aspects of the performance of mice of the B x D Ty RI strain series in the Morris navigation task. Behav Genet 32: 69-78

Peirce JL, Chesler EJ, Williams RW, Lu L (2003) Genetic architecture of the mouse hippocampus: identification of gene loci with selective regional effects. Genes Brain Behav 2: 238-52

Philip VM, Duvvuru S, Gomero B, Ansah TA, Blaha CD, Cook MN, Hamre KM, Lariviere WR, Matthews DB, Mittleman G, Goldowitz D, Chesler EJ (2010) High-throughput behavioral phenotyping in the expanded panel of BXD recombinant inbred strains. Genes Brain Behav 9: 129-159

Phillips TJ, Belknap JK, Crabbe JC (1991) Use of recombinant inbred strains to assess vulnerability to drug abuse at the genetic level. J Addict Dis 10: 73-87

Phillips TJ, Huson M, Gwiazdon C, Burkhart-Kasch S, Shen EH (1995) Effects of acute and repeated ethanol exposures on the locomotor activity of BXD recombinent inbred mice. Alcohol Clin Exp Res 19: 269-278

Phillips TJ, Lessov CN, Harland RD, Mitchell SR (1996) Evaluation of potential genetic associations between ethanol tolerance and sensitization in BXD/Ty recombinant inbred mice. J Pharmacol Exp Ther 277: 613-623

Quock RM, Mueller JL, Vaughn LK, Belknap JK (1996) Nitrous oxide antinociception in BXD recombinant inbred mouse strains and identification of quantitative trait loci. Brain Res 725: 23-9

Seecharan DJ, Kulkarni AL, Lu L, Rosen GD, Williams RW (2003) Genetic control of interconnected neuronal populations in the mouse primary visual system. J Neurosci 23: 11178-88

Wehner JM, Sleight S, Upchurch M (1990) Hippocampal protein kinase C activity is reduced in poor spatial learners. Brain Res 523: 181-7

Williams RW, Airey DC, Kulkarni A, Zhou G, Lu L (2001) Genetic dissection of the olfactory bulbs of mice: QTLs on four chromosomes modulate bulb size. Behav Genet 31: 61-77

Yang RJ, Mozhui K, Karlsson RM, Cameron HA, Williams RW, Holmes A (2008) Variation in mouse basolateral amygdala volume is associated with differences in stress reactivity and fear learning. Neuropsychopharmacology 33: 2595-604
